# Synthesis, Antiphospholipase A_2_, Antiprotease, Antibacterial Evaluation and Molecular Docking Analysis of Certain Novel Hydrazones

**DOI:** 10.3390/molecules21121664

**Published:** 2016-12-02

**Authors:** Nahed N. E. El-Sayed, Ahmed M. Alafeefy, Mohammed A. Bakht, Vijay H. Masand, Ali Aldalbahi, Nan Chen, Chunhai Fan, Abir Ben Bacha

**Affiliations:** 1 Department of Chemistry, College of Science, “Girls Section”, King Saud University, P.O. Box 22452, Riyadh 11495, Saudi Arabia; 2 National Organization for Drug Control and Research, Giza 35521, Egypt; 3 Department of Chemistry, Kulliyyah of Science, International Islamic University, P.O. Box 141, 25710 Kuantan, Malyasia; ahmed.alafeefy@gmail.com; 4 Department of Pharmaceutical Chemistry, College of Pharmacy, Prince Sattam Bin Abdulaziz University, P.O. Box 173, Al-kharj 11942, Saudi Arabia; bakhtpharm@gmail.com; 5 Department of Chemistry, Vidya Bharati College, Camp, Amravati, Maharashtra 444 602, India; vijaymasand@gmail.com; 6 Chemistry Department, King Saud University, P.O. Box 2455, Riyadh 11451, Saudi Arabia; aaldalbahi@ksu.edu.sa; 7 Division of Physical Biology & Bioimaging Center, Shanghai Synchrotron Radiation Facility, Shanghai Institute of Applied Physics, Chinese Academy of Sciences, Shanghai 201800, China; chennan@sinap.ac.cn (N.C.); fchh@sinap.ac.cn (C.F.); 8 Biochemistry Department, Science College, King Saud University, P.O. Box 22452, Riyadh 11495, Saudi Arabia; aalghanouchi@ksu.edu.sa

**Keywords:** benzo[*d*][1,3]oxazin-4-one, aroylhydrazides, *N*-acylhydrazones, hydrazones, benzylidene hydrazides, phospholipases, proteases, antimicrobial evaluation, molecular docking

## Abstract

Some novel hydrazone derivatives **6a**–**o** were synthesized from the key intermediate 4-Chloro-*N*-(2-hydrazinocarbonyl-phenyl)-benzamide **5** and characterized using IR, ^1^H-NMR, ^13^C-NMR, mass spectroscopy and elemental analysis. The inhibitory potential against two secretory phospholipase A_2_ (sPLA_2_), three protease enzymes and eleven bacterial strains were evaluated. The results revealed that all compounds showed preferential inhibition towards *h*GIIA isoform of sPLA_2_ rather than DrG-IB with compounds **6l** and **6e** being the most active. The tested compounds exhibited excellent antiprotease activity against proteinase K and protease from *Bacillus* sp. with compound **6l** being the most active against both enzymes. Furthermore, the maximum zones of inhibition against bacterial growth were exhibited by compounds; **6a**, **6m**, and **6o** against *P. aeruginosa*; **6a**, **6b**, **6d**, **6f**, **6l**, **6m**, **6n**, and **6o** against *Serratia*; **6k** against *S. mutans*; and compounds **6a**, **6d**, **6e**, **6m**, and **6n** against *E. feacalis.* The docking simulations of hydrazones **6a**–**o** with GIIA sPLA_2_, proteinase K and hydrazones **6a**–**e** with glutamine-fructose-6-phosphate transaminase were performed to obtain information regarding the mechanism of action.

## 1. Introduction

Hydrazones bearing azomethine moiety can be formed by condensation of hydrazides or aroyl hydrazides with aldehydes [1,2,3,4]. These compounds are most widely used as building blocks for the synthesis of 3-acetyl-2,5-disubstituted-2,3-dihydro-1,3,4-oxadiazoles [5], different mono-, di- and trisubstituted hydrazines [6,7], azetidin-2-ones, thiazolidin-4-ones and methyl thiazolidines [8,9], and formazan derivatives [10,11]. Hydrazones are well known to exhibit a wide spectrum of biological activities [12] and have been utilized as useful candidates for development of antimalarial [13], antitumor [14], antiviral [15,16], antimicrobial [17], antioxidant [18], analgesic, anti-inflammatory and anti-ulcerogenic agents [19,20].

Secreted phospholipases A_2_ (sPLA_2_s) are enzymes found in mammals and animal venoms that catalyze hydrolysis of glycerophospholipids at the *sn*-2 ester position to release a free fatty acid and a lysophospholipid [21]. Mammalian phospholipases have been categorized into ten groups IB, IIA, IIC, IID, IIE, IIF, III, V, X and XIIA [22,23]. It has been reported that sPLA_2_s play a key role in a good number of bio-chemical processes, thus, on one the hand, group IB sPLA_2_ has been proposed to be involved in various physiological and pathophysiological processes such as cellular proliferation, cell migration, hormone release [24] and apoptosis in neuronal cells [25]. In addition, higher levels of this enzyme were detected in the serum of patient with chronic renal failure [26] and acute lung injury [27] compared to healthy controls.

On the other hand, the GIIA sPLA_2_ has been correlated to inflammatory diseases, which is due to elevated levels of this enzyme were detected in the fluids of patients suffering from various inflammatory diseases such as rheumatoid arthritis [28], acute lung injury-acute respiratory distress syndrome (ALI-ARDS) [29] and pancreatitis-associated adrenal injury in acute necrotizing pancreatitis [30], as well as in various cancers [31]. Therefore, seeking potent and selective inhibitors to this sPLA_2_ may be considered an effective approach to develop novel anti-inflammatory and antitumor agents.

Proteases or proteinases enzymes are proteolytic enzymes that break down proteins into peptides and amino acids. They are classified into different classes depending on their source and functions [32] such as proteinase K, protease from *Bacillus* and protease-esperase. Biologically, proteases are involved in controlling many biological pathways in the life cycle of human, plants, animals, insects and pathogens such as fungi, bacteria and viruses. Many studies reported their involvement in the pathogenic processes of human diseases such as bleeding disorders [33], inflammation [34], cancer [35], hypertension [36] and AIDS [37]. Therefore, proteases inhibitors’ can be considered as targets in drug design for developing therapeutics and prevention of diseases [38].

In continuation to our previous efforts towards synthesis of novel biologically active compounds [39,40,41], we report herein the synthesis of novel hydrazones **6a**–**o** and evaluation of their phospholipases, proteases and bacterial inhibitory activities. The docking simulations of hydrazones **6a**–**o** against GIIA sPLA_2_, proteinase K and hydrazones **6a**–**e** against glutamine-fructose-6-phosphate transaminase were performed in order to obtain information regarding the mechanism of action.

## 2. Results and Discussion

### 2.1. Chemistry

**Scheme 1:**
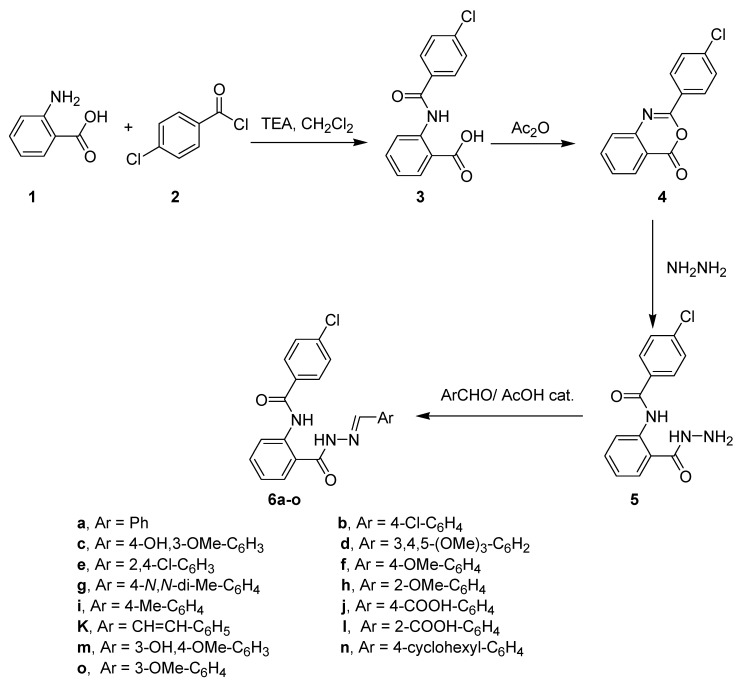
The synthesis of Schiff bases **6a**–**o**.

Benzoylation of anthranillic acid **1** with 4-chlorobenzoyl chloride **2** in methylene chloride in the presence of triethylamine afforded the corresponding amido acid **3** which upon boiling with excess of acetic anhydride underwent intramolecular cyclization and afforded benzo[*d*][1,3]oxazin-4-one derivative **4**. Nucleophilic attack by the amino group of hydrazine hydrate on the carbonyl group of the latter benzoxazinone, and subsequent ring opening gives the key intermediate 4-Chloro-*N*-(2-hydrazinocarbonyl-phenyl)-benzamide **5**. Condensation of hydrazino derivative **5** with a variety of aromatic aldehydes yielded the desired hydrazones **6a**–**o** (Scheme 1). The structures of these products were confirmed based on their spectral data and elemental analyses. In general, the IR spectra of compounds **6a**–**o** displayed two absorption bands at ν_max_ 3421–3292 cm^−1^, which are attributed to the two (NH) groups, and two absorption bands at ν_max_ 1684–1657 cm^−1^ due to the two carbonyl groups. In addition, the (C=N) groups appeared around 1598–1579 cm^−1^. Moreover, the ^1^H-NMR spectra of these compounds indicated the disappearance of the spectral line due to NH_2_ group of the starting hydrazide **5** and appearance of two broad singlet signals at δ 12.37–11.08 ppm corresponding to 2NH groups, singlet signals at δ 8.84–8.30 ppm due to the characteristic azomethines’ protons (N=CH) in addition to signals attributed to the aliphatic and aromatic protons at the expected chemical shift values. Analogously, the ^13^C-NMR spectra proved the presence the two C=O groups at δ_C_ 165.61–163.86 ppm, azomethine carbons’ at 151.46–145.10 ppm and the aliphatic and aromatic carbons at the expected chemical shift values. The mass spectra of all of the synthesized compounds revealed the existence of the parent ion peaks. Finally, the C, H, N elemental analyses of all of the synthesized compounds were in agreement with the proposed structures.

### 2.2. Biological Evaluation

#### 2.2.1. Phospholipases Inhibitory Activity

**Figure 1:**
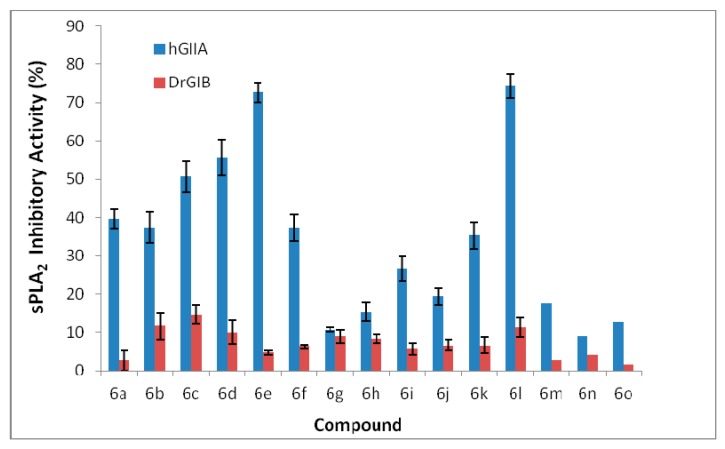
Inhibitory activity (%) of compounds **6a**–**o** against sPLA_2_ (hG-IIA and DrG-IB).

The phospholipase inhibitory activity of hydrazones **6a**–**o** have been detected against two different classes of sPLA_2_s, namely human group IIA sPLA_2_ (hG-IIA) and dromedary group IB sPLA_2_ (DrG-IB). The obtained results revealed that compounds **6c**, **6d**, **6e** and **6l** exhibited selective inhibition against *h*GIIA rather than DrG-IB by 50.67% ± 4.04%, 55.67% ± 4.72%, 72.67% ± 2.51% and 74.33% ± 3.21%, respectively, as shown in Figure 1 and Table S1.

Conversely, the lowest inhibitory activity against this enzyme (9% ± 2%) was displayed by compound **6n**. These results are in agreement with the data obtained in our previous work [42].

As many clinical studies revealed that the levels of sPLA_2_s are increased in different inflammatory conditions such as rheumatoid arthritis [28], these active hydrazones can be proposed as potential candidates to explore their utility for such ailment.

#### 2.2.2. Proteases Inhibitory Activity

**Figure 2:**
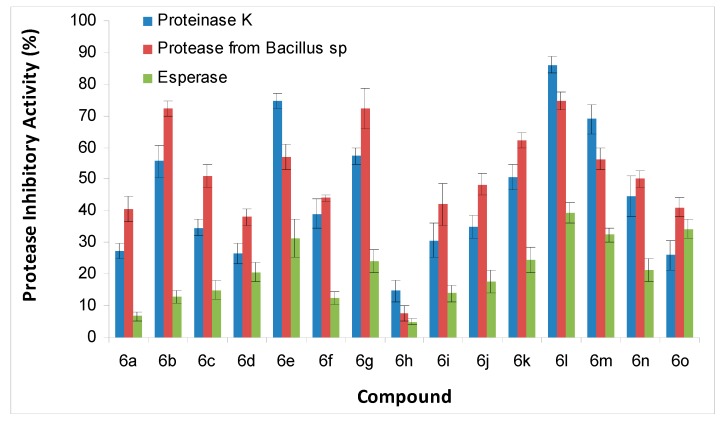
Antiprotease activity (%) of compounds **6a**–**o** against different protease enzymes.

The synthesized compounds were also screened for in vitro anti-proteases activity against the commercially available protease-esperase, proteinase K and protease obtained from *Bacillus* sp. The screening results shown in Figure 2 and Table S2 revealed that the tested compounds displayed varied degrees of proteases inhibition. The maximum inhibitory activities against proteinase K (86 ± 2.64), protease from *Bacillus* (74.66 ± 2.88) and protease-esperase (39.33 ± 3.21) were exhibited by compound **6l**.

Furthermore, the next highest inhibitory activities against proteinase K were exhibited by compounds **6e** (74.66% ± 2.51%) and **6m** (69% ± 4.58**%**), while the next highest inhibitory activities against protease from *Bacillus* sp. were exhibited by compounds **6b** (72.33% ± 2.51%) and **6g** (72.33% ± 6.42%).

It is worth mentioning that most of the compounds showed good antiprotease inhibition against proteinase K and protease from *Bacillus* sp., as depicted in Figure 2. It was also noticed that there was alignment in inhibitory activity against these two enzymes, as any single compound that showed high inhibitory potential against one of them also reacted in a similar fashion towards the other one.

As the anti-inflammatory activities of chemical compounds can be expressed by phospholipase A_2_ (hGIIA) and/or through protease inhibitor potentials [42], compound **6l**, which was found to be the most active candidate against both phospholipases A_2_ (sPLA_2_) and protease enzymes under investigation, may be proposed as promising potential anti-inflammatory agent for treatment of ulcerative colitis.

#### 2.2.3. Antibacterial Screening

Finally, compounds **6a**–**o** were further examined for in vitro antibacterial activity. Preliminary screening against eleven strains of Gram-positive and Gram-negative bacteria was performed by adopting standard protocol [43]. The antibacterial potency was determined by measuring the inhibition zones. All tests were performed in duplicates and means of inhibition zones were recorded in mm as presented in Table 1.

Analysis of the screening data revealed that the inhibition activity produced by some of the tested compounds was found to be good to excellent compared to the used reference drug tetracycline. Among the eleven strains that would be considered more susceptible to inhibition by one or more of the synthetic compounds were; *P. aeruginosa*, *Serratia*, *S. aureus*, *S. mutans* and *E. feacalis* with genus *Serratia* being the most sensitive one which was inhibited by nine hydrazones. The highest inhibition towards this pathogen was displayed by compounds **6a**, **6b**, **6d**, **6e**, **6f**, **6l**, **6m**, **6n**, and **6o** with inhibition zones of 15.5 ± 0.0, 12.5 ± 0.0, 12 ± 0.0, 14 ± 1.0, 14 ± 1.0, 13 ± 0.01, 14.5 ± 1.5, 14.5 ± 0.5 and 12.5 ± 0.5, respectively. The highest inhibitory activity against *P. aeruginosa* was exerted by compounds **6a**, **6m**, and **6o** with inhibition zones of 17 ± 1.0, 18.5 ± 0.5 and 18.5 ± 0.5, respectively. Although compounds **6j**, **6l** and **6n** produced the highest inhibition zones against *S. aureus* strain, they were considered to be less effective compared to the reference drug tetracycline. Moreover, the maximum zone of inhibition (24.5 ± 0.5) was exhibited by compound **6k** against *S. mutans*. Finally, the reference drug tetracycline was found to be completely inactive against *E. feacalis*, while compounds **6a**, **6d**, **6e**, **6m**, and **6n** exhibited the maximum inhibition zones of 18.5 ± 0.5, 18.0. ± 1.0, 18.5 ± 0.5, 13.5 ± 1.5 and 18.5 ± 0.5, respectively. The rest of the compounds showed moderate inhibition against all other bacterial strains.

### 2.3. Molecular Docking Analysis

Based on the data obtained from different biological evaluations, compounds **6a**–**o** were docked against proteinase K, GIIA sPLA_2_, and compounds **6a**–**e** against glutamine-fructose-6-phosphate transaminase (GlcN6P) synthase (GlmS, l-glutamine: d-fructose-6P amidotransferase, EC 2.6.1.16) in order to provide a conceivable rationale for the observed activities.

#### 2.3.1. Docking Simulations for Compounds **6a**–**o** in Active site of GIIAsPLA_2_

GIIAsPLA_2_ is a low molecular weight enzyme (14 kDa) with seven disulfide bonds with a highly conserved Ca^2+^-binding loop and a catalytic dyad consisting of His47/Asp91 along with active a hydrophobic region lined near the *N*-terminal helix [44].

**Figure 3:**
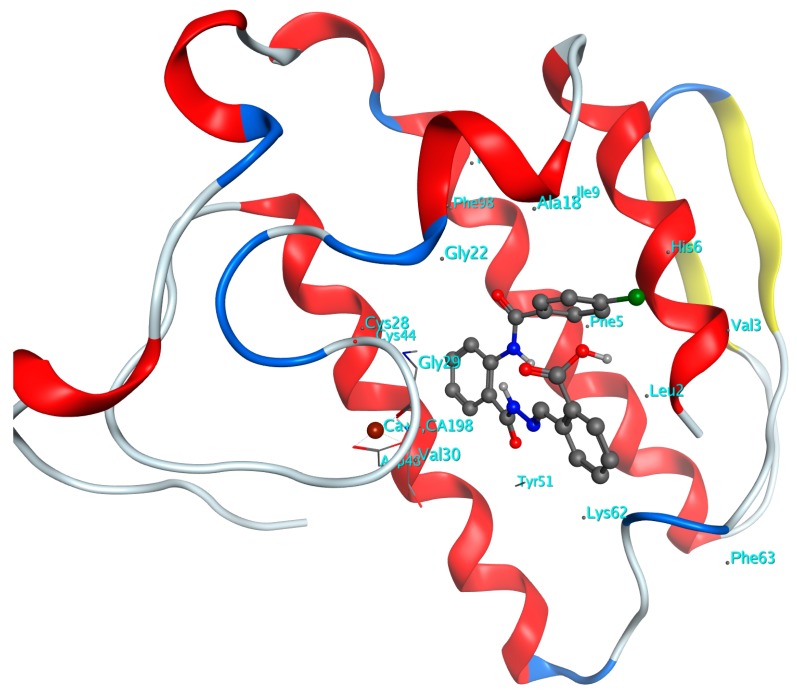
Docking pose for compound **6l** as a representative, in the active site of GIIAsPLA_2_.

The docking simulations for **6a**–**o** in active site of GIIAsPLA_2_ are presented in Supplementary Materials, Figure S1. Results of docking simulations for compound **6l**, the most active anti-GIIAsPLA_2_ enzyme, depicted here as a representative in Figure 3, revealed that it interacts with hydrophobic and polar residues of active site of GIIAsPLA_2_. Compound **6l** has adopted “U” shape with its central benzene ring oriented towards the polar residues viz. His6, Gly22, Asp48, and Val30. The –CONH–N– moiety is responsible for interactions with Phe5 and His47. The –CO–NH–N= moiety bridging the two benzene rings as a polar linker has incorporated good flexibility to the ligand, hence such a flexible moiety is beneficial for further amendments. The –Cl and –COOH groups are oriented toward Leu2 and Val3 due to polar interactions. The –CO– group of –CONH– group is close to Gly22. Hence, –CONH– group is important group for retention in future optimizations.

#### 2.3.2. Docking Simulations for Compounds **6a**–**o** in Active Site of Proteinase K

Proteinase K (EC 3.4.21.64, protease K, endopeptidase K, Tritirachium alkaline proteinase, Tritirachium album serine proteinase, and Tritirachium album proteinase K) is a broad-spectrum serine protease belonging to Peptidase family S8 with ability to digest proteins. It is used for the destruction of proteins in cell lysates (tissue, and cell culture cells) and for the release of nucleic acids [45].

**Figure 4:**
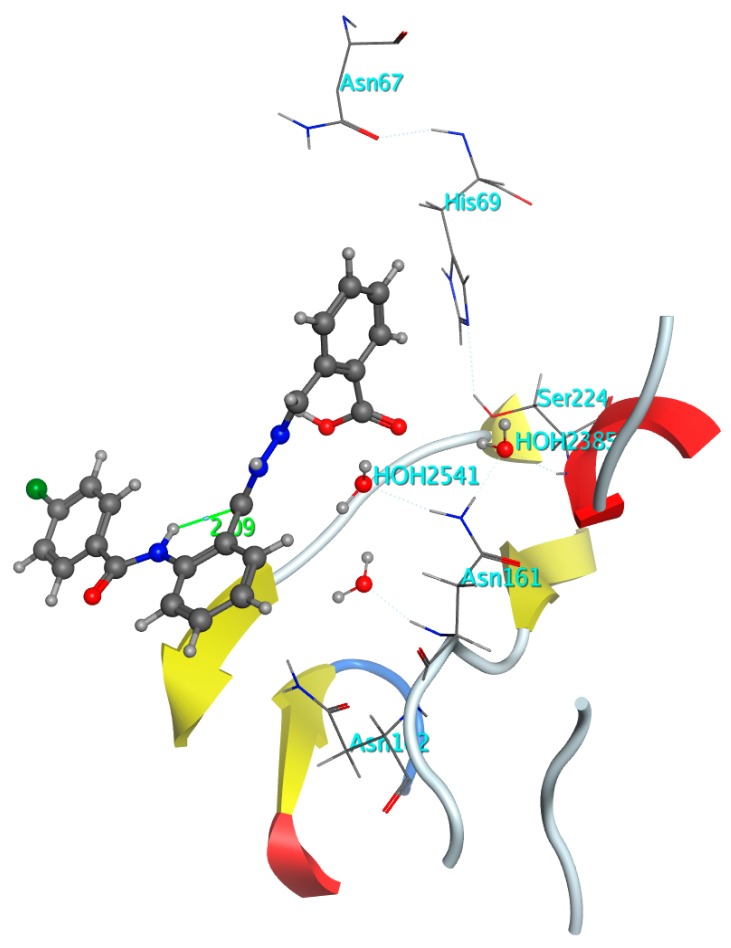
Docking pose for compound **6l** as a representative, in the active site of proteinase K.

The docking simulations for hydrazones **6a**–**o** in active site of proteinase K are presented in Supplementary Materials, Figure S2. Results of docking simulations for compound **6l**, the most active antiproteinase K, depicted here as a representative in Figure 4, revealed that it interacts with hydrophobic and polar residues of active site of proteinase K. Compound **6l** is unable to occupy the complete space of active site of the enzyme. It interacts with polar residues viz. Asn161, Asn162, Ser224 and His69. In addition, it has H-bond formation with H_2_O (at distance of 2.86 A°), present inside the active site of Proteinase K, due to the –COOH group present on benzene ring. Hence, the –COOH group is beneficial. Another important interaction is arene-cation interaction between the benzene ring possessing –Cl atom with H_2_O (at distance of 4.08 A°), present inside the active site of proteinase K. Interestingly, the compound possesses intramolecular H-bond formation (distance 2.09 A°) between the –NH– part of –CONH– group with –CO– part of –CONH–N– group. This H-bond is probably responsible for the specific orientation and shape of the molecule inside the active site. The compound **6l** has acquired weird “J” or “L” shape inside the active site. The benzene ring possessing –COOH group is closer to the active site residues. This indicates that the –CONH– and –CONH–N– groups are important for future modifications.

#### 2.3.3. Docking Simulations for Compounds **6a**–**e** in Active Site of Glutamine-Fructose-6-Phosphate Transaminase

The molecular mechanism of multifarious reaction catalyzed by glucosamine-6-phosphate (GlcN6P) synthase (GlmS, l-glutamine: d-fructose-6P amidotransferase, EC 2.6.1.16) enzyme comprises both ammonia transfer (l-glutamine to Fru-6-P) and sugar isomerization (fructosamine-6-phosphate to glucosamine-6-phosphate). This reaction is the initiation of the pathway leading to the eventual production of uridine 5′-diphospho-*N*-acetyl-d-glucosamine (UDP-GlcNAc), a product that is present in all class of organisms, but in fungi and bacteria it is utilized to construct macromolecules essential for the cell wall assembly, such as a number of amino sugar-containing macromolecules, comprising chitin and mannoproteins in fungi, and peptidoglycan and lipopolisaccharides in bacteria. In the prokaryotic cell, the inhibition of GlcN6P synthase even for a small time is fatal. Fortunately, because of the longer lifespan of human, the running down of amino sugar pool for a short time is not deadly. Consequently, it has been proposed as a possible target for developing antibacterial and antifungal agents [46,47,48,49].

It is evident that recognition of a molecule by a particular enzyme is dependent on the structural properties of that molecule and its distribution of the molecular electrostatic potential (MEP). Furturmore, analysis of ligand–receptor interactions for GlcN-6-P synthase revealed that ligands having primary amido groups can form stable hydrogen bonds with the amino acids residues present in the binding site of the fungal enzyme thus they may serve as “anchors” to lock the inhibitor in the binding pocket of the enzyme. In addition, previous structure–activity relationship experiments revealed that presence of active electrophilic center at a proper position is required for inactivation of the enzyme to take place [49]. By analogy, hydrazones **6a**–**o** having the same structural features represented by two primary amido groups and electrophilic double bond may serve as inhibitors for bacterial GlcN6P synthase. This hypothesis is examined by docking hydrazones **6a**–**e** in the active site of the bacterial enzyme (these results are presented in Supplementary Materials, Figure S3).

**Figure 5:**
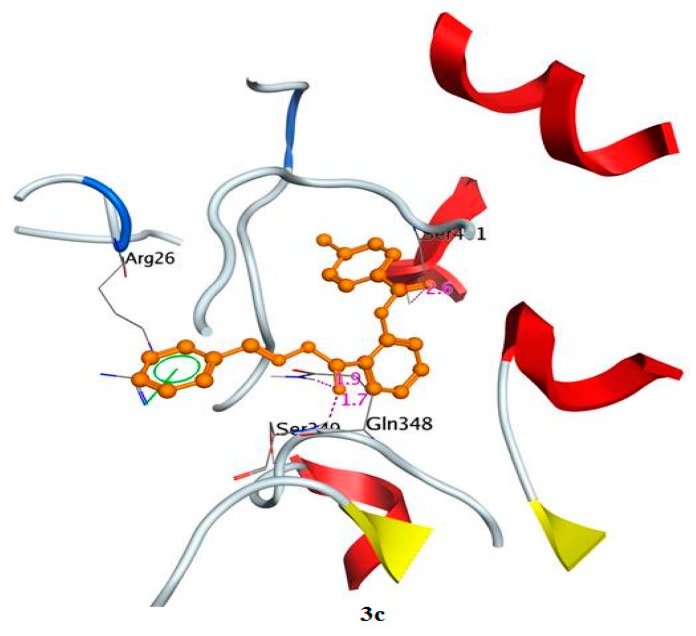
Docking pose for compound **6a**, as a representative, in the active site of glucosamine-6-phosphate (GlcN6P) synthase.

Docking simulations for the most active candidate **6a** which inhibits collectively three bacterial strains and exhibiting inhibition zones larger than those produced by the reference drug tetracycline against two bacterial strains, *Serratia* and *E. feacalis*, depicted here as a representative in Figure 5, revealed that it interacts with a good number of residues of active site of glucosamine-6-phosphate (GlcN6P) synthase. It mainly interacts with polar and hydrophobic residues of the active site. It has adopted weird “J” or “L” shape in the active site. The oxygen atoms of the two amide groups are responsible for the formation of H-bonding with the polar residues Ser A401 (distance 2.61 A°), Gln A348 (distance 1.94 A°) and Ser A349 (distance 1.71 A°). Hence, the amide groups are beneficial for future development. The arene-cation interaction of benzene ring of ligand with the polar residue Arg A26 has strengthened the binding of ligand with the receptor. The chlorine atom on the benzene ring of the ligand has hydrophobic interactions with the hydrophobic residues Trp A74, Ala A602 and Val A399 that constitute the lipophilic region of the cavity. The –CO-NH-N– moiety present between the two benzene rings as a polar linker has furnished high flexibility to the ligand, hence such a flexible moiety is advantageous for further modifications. The benzene ring, which is acting as a bridge between the two –CONH– groups, is oriented toward polar region of the active site, which comprises polar residues such as Ser A401, Cys A300, Ser A303, Ser A347, Thr A352, Gln A348 and Ser A349.

## 3. Experimental Section

### 3.1. Chemistry

#### 3.1.1. General

All the chemicals were purchased from various suppliers, and were used without further purification, unless otherwise stated. Melting points were measured on a Gallenkamp melting point apparatus (Sanyo Gallenkamp, South borough, UK) in open glass capillaries and are uncorrected. Infrared spectra (IR) were recorded using the KBr disc technique using a Perkin Elmer FT-IR spectrophotometer 1000 (PerkinElmer, Waltham, MA, USA). ^1^H- and ^13^C-NMR spectra were recorded on a BRUCKER-PLUS NMR (Billerica, MA, USA) operating at 500 MHz in deuterated dimethyl sulfoxide (DMSO-*d*_6_). Chemical shifts are referred to in terms of ppm and *J*-coupling constants are given in Hz. Mass Spectra were recorded on a Shimadzu GCMS-QP 5000 instrument (Shimadzu, Tokyo, Japan). Elemental analysis was done to evaluate the presence of C, H, and N by Perkin Elmer-series-II and the found results were within ±0.4% of the theoretical values. The biological evaluations of the products were carried out at King Saud University, Riyadh, KSA.

#### 3.1.2. Synthetic Procedures

Compounds **3** and **4** were prepared according to reported procedures [20].

##### 4-Chloro-*N*-(2-(hydrazinecarbonyl)phenyl)benzamide **5**

A mixture of 2-(4-chlorophenyl)-4*H*-benzoyl [*d*][1,3]oxazin-4-one **4** (2.57 g, 10 mmol) and 10 mL of hydrazine hydrate (80%) was refluxed for 1 h. Evaporation of the excess hydrazine under reduced pressure and washing of the remaining solid product with plenty of water afford the title compound in pure form.

Yield (95%); shiny white powder; m.p. 175–177 °C. IR ( KBr) ν_max_: 3316–3128 (NH_2_ and 2NH), 1673, 1635 (2 C=O), 1592 (C=N) cm^−1^; ^1^H-NMR (DMSO-*d*_6_) δ: 12.04 (s, 1H, NHCO), 11.92 (s, 1H, NHCO), 8.51 (d, 1H, Ar-H, *J* = 8.3 Hz), 7.95 (d, 2H, 2 × Ar-H, *J* = 8.4 Hz), 7.90 (d, 1H, Ar-H, *J* = 7.6 Hz), 7.76–7.62 (m, 3H, 3 × Ar-H), 7.28 (t, 1H, Ar-H, *J* = 7.5 Hz), 4.52 (s, 2H, NH_2_); ^13^C-NMR (DMSO-*d*_6_) δ: 165.27 (CO), 164.16 (CO), 138.2, 137.10, 132.43, 129.11, 128.72, 127.80, 125.445, 124.32, 120.93 (12 × Ar-C); MS (ESI): 290 [M^+^ + 1], Anal. Calcd. For C_14_H_12_ClN_3_O_2_: C (58.04%); H (4.17%); N (14.50%); Found: C (58.08%); H (4.21%); N (14.53%).

##### Synthesis of Compounds **6a**–**o**

General procedures: A mixture of 4-chloro-*N*-(2-(hydrazinecarbonyl)phenyl)benzamide **5** (2.89 g, 10 mmol) and the appropriate aldehyde (10 mmol) was refluxed in absolute ethanol (15 mL) in presence of few drops of glacial acetic acid as a catalyst for about 4 hour and monitored with TLC (*n*-hexane:EtOAc, 80:20) until the reaction was completed as observed by appearance of a single new spot. The resultant reaction mixture was cooled to room temperature and the solid product obtained in each experiment was collected by filtration and recrystallized from ethanol to afford the desired products **6a**–**o**

*N-(2-(2-benzylidenehydrazine-1-carbonyl)phenyl)-4-chlorobenzamide* (**6a**) Yield (89%); white crystals; m.p. 244–246 °C; IR (KBr) ν_max_: 3336–3197 (2 NH), 1673, 1635 (2 C=O), 1592 (C=N) cm^−1^; ^1^H-NMR (DMSO-*d*_6_) δ: 12.14 (s, 1H, NHCO), 11.94 (s, 1H, NHCO), 8.51 (d, 1H, Ar-H, *J* = 8.3 Hz), 8.47 (s, 1H, N=CH), 7.96 (d, 2H, 2 × Ar-H, *J* = 8.4 Hz), 7.92 (d, 1H, Ar-H, *J* = 7.6 Hz), 7.76–7.47 (m, 8H, 8 × Ar-H), 7.30 (t, 1H, Ar-H, *J* = 7.5 Hz); ^13^C-NMR (DMSO-*d*_6_) δ: 165.39 (CO), 164.00 (CO), 149.53 (N=CH), 139.53, 137.42, 134.49, 133.63, 133.06, 130.86, 129.48, 129.36, 129.10, 127.75, 123.85, 121.85, 121.75, 121.31 (18 × Ar-C); MS (ESI): 379 (C_21_H_16_ClN_3_O_2_, [M^+^ + 1]; Anal. Calcd. for C_21_H_16_ClN_3_O_2_: C (66.76%); H (4.27%); N (11.12%); Found: C (66.72%); H (4.24%); N (11.14%).

*4-Chloro-N-(2-(2-(4-chlorobenzylidene)hydrazine-1-carbonyl)phenyl)benzamide* (**6b**) Yield (95%); white powder; m.p. 252–256 °C; IR (KBr) ν_max_: 3333–3201 (2 NH), 1675, 1639 (2 C=O), 1598 (C=N) cm^−1^; ^1^H-NMR (DMSO-*d*_6_) δ: 12.19 (s, 1H, NHCO), 11.95 (s, 1H, NHCO), 8.52 (d, 1H, Ar-H, *J* = 8.3 Hz), 8.45 (s, 1H, N=CH), 7.96 (d, 2H, 2 × Ar-H, *J* = 8.4 Hz), 7.90 (d, 1H, Ar-H, *J* = 7.8 Hz), 7.77 (d, 2H, 2 × Ar-H, *J* = 8.4 Hz), 7.65–7.61 (m, 3H, 3 × Ar-H), 7.52 (d, 2H, 2 × Ar-H, *J* = 8.4 Hz), 7.28 (t, 1H, Ar-H, *J* = 7.6 Hz); ^13^C-NMR (DMSO-*d*_6_) δ: 165.46 (CO), 163.96 (CO), 148.13 (N=CH), 139.62, 137.43, 135.32, 133.58, 133.42, 133.10, 129.67, 129.44, 129.33, 129.07, 123.76, 121.71, 121.08 (18 × Ar-C); MS (ESI): 413 [M^+^ + 1], Anal. Calcd. for C_21_H_15_Cl_2_N_3_O_2_: C (61.18%); H (3.67%); N (10.19%); Found: C (61.20%); H (3.64%); N (10.21%).

*4-Chloro-N-(2-(2-(4-hydroxy-3-methoxybenzylidene)hydrazine-1-carbonyl)phenyl)benzamide* (**6c**) Yield (85%); white crystals; m.p. 236–238 °C; IR (KBr) ν_max_: 3341–3201 (2 NH and OH), 1675, 1639 (2 C=O), 1588 (C=N) cm^−1^; ^1^H-NMR (DMSO-*d*_6_) δ 11.93 (s, 2H, NH and OH), 9.59 (br. s, 1H, NH), 8.46 (d, 1H, Ar-H, *J* = 8.3 Hz), 8.30 (s, 1H, N=CH), 7.90 (d, 2H, 2 × Ar-H, *J* = 8.4 Hz), 7.83 (d, 1H, Ar-H, *J* = 7.7 Hz), 7.58 (d, 2H, 2 × Ar-H, *J* = 8.4 Hz), 7.54 (d, 1H, Ar-H, *J* = 7.7 Hz), 7.29 (d, 1H, Ar-H *J* = 1.0 Hz), 7.21 (t, 1H, Ar-H, *J* = 7.7 Hz), 7.06 (dd, 1H, Ar-H, *J* = 8.1, 1.0 Hz), 6.80 (d, 1H, Ar-H, *J* = 8.1 Hz), 3.78 (s, 3H, OCH_3_); ^13^C-NMR (DMSO-*d*_6_) δ 165.13 (CO), 163.98 (CO), 150.24, 149.81, 148.55, 139.52, 137.41, 133.65, 132.90, 129.46, 129.01, 125.84, 123.78, 123.06, 121.63, 121.31, 115.93, 109.49 (CH=N and 18 × Ar-C), 56.04 (OCH_3_); MS (ESI): 425 [M^+^ + 1], Anal. Calcd. for C_22_H_18_ClN_3_O_4_: C (62.34%); H (4.28%); N (9.91%); Found: C (62.32%); H (4.24%); N (9.93%).

*4-Chloro-N-(2-(2-(3,4,5-trimethoxybenzylidene)hydrazine-1-carbonyl)phenyl)benzamide* (**6d**) Yield (93%); white crystals; m.p. 247–248 °C; IR (KBr) ν_max_: 3345–3208 (2 NH), 1671, 1644 (2 C=O), 1578 (C=N) cm^−1^; ^1^H-NMR (DMSO-*d*_6_) δ: 12.11 (s, 1H, NHCO), 11.81 (s, 1H, NHCO), 8.46 (d, 1H, Ar-H, *J* = 8.2 Hz), 8.37 (s, 1H, N=CH), 7.96 (d, 2H, 2 × Ar-H, *J* = 8.4 Hz), 7.88 (d, 1H, Ar-H, *J* = 7.6 Hz), 7.67 (d, 2H, 2 × Ar-H, *J* = 8.4 Hz), 7.63 (t, 1H, Ar-H, *J* = 8.2 Hz), 7.31 (t, 1H, Ar-H, *J* = 7.6 Hz), 7.04 (s, 2H, 2 × Ar-H), 3.85 (s, 6H, 2 × OCH_3_), 3.74 (s, 3H, OCH_3_); ^13^C-NMR (DMSO-*d*_6_) δ: 165.25 (CO), 164.12 (CO), 153.69 (3 × C-OCH_3_), 146.48 (N=CH), 139.32, 137.39, 133.68, 129.97, 129.52, 129.47, 129.15, 123.96, 121.96, 104.96 (15 × Ar-C), 60.60 (OCH_3_), 56.50 (OCH_3_), 56.46 (OCH_3_). MS (ESI): 469 [M^+^ + 1], Anal. Calcd. for C_24_H_22_ClN_3_O_5_: C (61.61%); H (4.74%); N (8.98%); Found: C (61.32%); H (4.71%); N (9.02%).

*4-Chloro-N-(2-(2-(2,4-dichlorobenzylidene)hydrazine-1-carbonyl)phenyl)benzamide* (**6e**) Yield (93%); creamish powder; m.p. 262–264 °C; IR (KBr) ν_max_: 3421–3268 (2 NH), 1680, 1656 (2 C=O), 1592 (C=N) cm^−1^; ^1^H-NMR (DMSO-*d*_6_) δ: 12.36 (s, 1H, NHCO), 11.79 (s, 1H, NHCO), 8.82 (s, 1H, N=CH), 8.47 (d, 1H, Ar-H, *J* = 8.3 Hz), 8.04 (d, 1H, Ar-H, *J* = 8.5 Hz), 7.95 (d, 2H, 2 × Ar-H, *J* = 8.4 Hz), 7.91 (d, 1H, Ar-H, *J* = 7.8 Hz), 7.75 (s, 1H, CH-Ar), 7.69–7.64 (m, 3H, 3 × Ar-H), 7.55 (d, 1H, Ar-H, *J* = 8.5 Hz), 7.31 (t, 1H, Ar-H, *J* = 7.6 Hz); ^13^C-NMR (DMSO-*d*_6_) δ: 165.31 (CO), 164.42 (CO), 148.38 (N=CH), 139.64, 137.93, 135.26, 133.45, 133.39, 133.10, 129.51, 129.49, 129.27, 129.11, 123.80, 121.71, 121.88 (18 × Ar-C). MS (ESI): 447 [M^+^ + 1], Anal. Calcd. for C_21_H_14_Cl_3_N_3_O_2_: C (56.46%); H (3.16%); N (9.41%); Found: C (56.48%); H (3.20%); N (9.42%).

*4-Chloro-N-[2-(4-methoxy-benzylidene-hydrazinocarbonyl)-phenyl]-benzamide* (**6f**) Yield (88%); white powder; m.p. 260–262 °C; IR (KBr) *v_max_*: 3374–3242 (2 NH), 1682, 1646 (2 C=O), 1589 (C=N) cm^−1^; ^1^H-NMR (DMSO-*d*_6_) δ: 12.06 (s, 1H, NHCO), 12.03 (s, 1H, NHCO), 8.55 (d, 1H, Ar-H, *J* = 8.3 Hz), 8.42 (s, 1H, CH=N), 7.96 (d, 2H, 2 × Ar-H, *J* = 8.5 Hz), 7.91 (d, 1H, Ar-H, *J* = 7.6 Hz), 7.69 (d, 2H, 2 × Ar-H, *J* = 8.7 Hz), 7.64 (d, 2H, 2 × Ar-H, *J* = 8.5 Hz), 7.60 (t, 1H, Ar-H, *J* = 7.5 Hz), 7.27 (t, 1H, Ar-H, *J* = 7.5 Hz), 7.02 (d, 2H, 2 × Ar-H, *J* = 8.7 Hz), 3.80 (s, 3H, OCH_3_); ^13^C-NMR (DMSO-*d*_6_) δ: 165.25 (CO), 163.91 (CO), 161.55 (C-OCH_3_), 149.54 (N=CH), 139.65, 137.42, 133.60, 132.96, 129.45, 129.42, 128.99, 127.01, 123.69, 121.53, 121.02, 114.82 (17 × Ar-C), 55.75 (OCH_3_). MS (ESI): 409 [M^+^ + 1], Anal. Calcd. for C_22_H_18_ClN_3_O_3_: C (64.79%); H (4.45%); N (10.30%); Found: C (64.78%); H (4.42%); N (10.32%).

*4-Chloro-N-(2-(2-(4-(dimethylamino)benzylidene)hydrazine-1-carbonyl)phenyl)benzamide* (**6g**) Yield (82%); yellow powder; m.p. 249–251 °C; IR (KBr) ν_max_: 3364–3212 ((2 NH), 1679, 1640 (2 C=O), 1582 (C=N) cm^−1^; ^1^H-NMR (DMSO-*d*_6_) δ: 12.11 (s, 1H, NHCO), 11.85 (s, 1H, NHCO), 8.56 (d, 1H, Ar-H, *J* = 8.2 Hz), 8.32 (s, 1H, CH=N), 7.96 (d, 2H, 2 × Ar-H, *J* = 8.5 Hz), 7.90 (d, 1H, Ar-H, *J* = 7.2 Hz), 7.68 (d, 2H, 2 × Ar-H, *J* = 8.5 Hz), 7.62 (t, 1H, Ar-H, *J* = 7.4 Hz), 7.56 (d, 2H, 2 × Ar-H, *J* = 8.8 Hz), 7.28 (t, 1H, Ar-H, *J* = 7.4 Hz), 6.77 (d, 2H, 2 × Ar-H, *J* = 8.8 Hz), 2.99 (s, 6H, 2 × N-CH_3_); ^13^C-NMR (DMSO-*d*_6_) δ: 165.31 (CO), 164.13 (CO), 152.4 (C-NCH_3_), 148.27 (N=CH), 138.2, 137.4, 134.84, 129.53, 129.44, 129.18, 128.8, 127.7, 124.2, 123.74, 119.8, 112.3 (17 × Ar-C), 45.2 (2 × CH_3_). MS (ESI): 422 [M^+^ + 1], Anal. Calcd. for C_23_H_21_ClN_4_O_2_: C (65.63%); H (5.03%); N (13.31%); Found: C (65.59%); H (5.02%); N (13.33%).

*4-Chloro-N-(2-(2-(2-methoxybenzylidene)hydrazine-1-carbonyl)phenyl)benzamide* (**6h**) Yield (98%); white powder; m.p. 236–238 °C; IR (KBr) ν_max_: 3383–3251 ((2 NH), 1680, 1652 (2 C=O), 1589 (C=N) cm^−1^; ^1^H-NMR (DMSO-*d*_6_) δ: 12.06 (s, 1H, NHCO), 11.97 (s, 1H, NHCO), 8.77 (s, 1H, N=CH), 8.47 (d, 1H, Ar-H, *J* = 8.3 Hz), 7.90 (d, 2H, 2 × Ar-H, *J* = 8.5 Hz), 7.88–7.83 (m, 2H, 2 × Ar-H), 7.60 (d, 2H, 2 × Ar-H, *J* = 8.5 Hz), 7.58–7.20 (m, 3H, 3 × Ar-H, *J* = 7.5 Hz), 7.05 (d, 1H, Ar-H, *J* = 8.4 Hz), 6.98 (t, 1H, Ar-H, *J* = 7.7 Hz), 3.80 (s, 3H, O-CH_3_); ^13^C-NMR (DMSO-*d*_6_) δ: 165.28 (CO), 163.96 (CO), 158.39 (C-OCH_3_), 145.11 (N=CH), 139.63, 137.42, 133.64, 133.05, 132.40, 129.49, 129.46, 129.09, 126.09, 123.77, 122.47, 121.58, 121.23, 120.99, 112.37 (17 × Ar-C), 56.18 (OCH_3_). MS (ESI): 409 [M^+^ + 1], Anal. Calcd. for C_22_H_18_ClN_3_O_3_: C (64.79%); H (4.45%); N (10.30%); Found: C (64.82%); H (4.46%); N (10.33%).

*4-Chloro-N-(2-(2-(4-methylbenzylidene)hydrazine-1-carbonyl)phenyl)benzamide* (**6i**) Yield (91%); white crystals; m.p. 256–258 °C; IR (KBr) ν_max_: 3372-3264 ((2 NH), 1684, 1657 (2 C=O), 1579 (C=N) cm^−1^; ^1^H-NMR (DMSO-*d*_6_) δ: 12.08 (s, 1H, NHCO), 11.97 (s, 1H, NHCO), 8.52 (d, 1H, Ar-H, *J* = 8.3 Hz), 8.43 (s, 1H, N=CH), 7.98 (d, 1H, Ar-H, *J* = 8.5 Hz), 7.91 (d, 2H, 2 × Ar-H, *J* = 7.7 Hz), 8.68–7.62 (m, 5H, 5 × Ar-H), 7.31–7.28 (m, 3H, 3 × Ar-H), 2.34 (s, 3H, CH_3_); ^13^C-NMR (DMSO-*d*_6_) δ (ppm): 165.31 (CO), 163.98 (CO), 149.61 (N=CH), 140.77, 139.55, 137.42, 133.62, 133.62, 133.02, 131.78, 129.97, 129.49, 129.46, 129.06, 127.74, 123.81, 121.67, 121.24 (18 × Ar-C), 21.52 (CH_3_). MS (ESI): 393 [M^+^ + 1], Anal. Calcd. for C_22_H_18_ClN_3_O_2_: C (67.43%); H (4.63%); N (10.72%); Found: C (64.42%); H (4.66%); N (10.73%).

*4-((2-(2-(4-chlorobenzamido)benzoyl)hydrazono)methyl)benzoic acid* (**6j**) Yield (85%); creamish powder; m.p. 252–254 °C; IR (KBr) ν_max_: 3373–3292 (2 NH, OH), 1759, 1680, 1652 (3 C=O), 1581 (C=N) cm^−1^; ^1^H-NMR (DMSO-*d*_6_) δ: 12.25 (s, 1H, NHCO), 11.92 (s, 1H, NHCO), 8.51 (s, 1H, N=CH), 8.04–7.25 (m, 12H, 12 × Ar-H); ^13^C-NMR (DMSO-*d*_6_) δ: 167.37 (COOH), 165.58 (CONH), 163.98 (CO), 148.24 (N=CH), 139.63, 138.49, 137.44, 133.56, 133.13, 132.42, 130.26, 129.40, 129.30, 129.09, 127.72, 123.75, 121.75, 121.05 (18 × Ar-C). MS: *m*/*z* 423 [M^+^ + 1], Anal. Calcd. for C_22_H_16_ClN_3_O_4_: C (62.64%); H (3.82%); N (9.96%); Found: C (62.63%); H (3.86%); N (9.97%).

*Chloro-N-(2-(2-(3-phenylallylidene)hydrazine-1-carbonyl)phenyl)benzamide* (**6k**) Yield (93%); creamish powder; m.p. 248–250 °C; IR (KBr) ν_max_: 3391–3271 (2 NH), 1680, 1644 (2 C=O), 1594 (C=N) cm^−1^; ^1^H-NMR (DMSO-*d*_6_) δ: 12.04 (s, 1H, NHCO), 11.97 (s, 1H, NHCO), 8.51 (d, 1H, Ar-H, *J* = 7.8 Hz), 8.25 (d, 1H, N=CH, *J* = 8.3 Hz), 7.95 (d, 2H, 2 × Ar-H, *J* = 8.5 Hz), 7.88 (d, 1H, *J* = 7.8 Hz, Ar-H), 7.68 (d, 2H, 2 × Ar-H, *J* = 8.5 Hz), 7.64–7.62 (m, 3H, 3 × Ar-H, *J* = 7.6 Hz), 7.41 (t, 2H, 2 × Ar-H, *J* = 7.3 Hz), 7.36–7.08 (m, 4H, 4 × Ar-H); ^13^C-NMR (DMSO-*d*_6_) δ: 165.29 (CO), 163.92 (CO), 151.46, 140.39, 139.49, 137.44, 136.26, 129.50, 129.45, 129.34, 129.05, 127.67, 125.84, 123.84, 121.28 (N=CH-CH=CH and 18 × Ar-C). MS: *m*/*z* 405 [M^+^ + 1], Anal. Calcd. for C_23_H_18_ClN_3_O_2_: C (68.40%); H (4.49%); N (10.40%); Found: C (68.42%); H (4.51%); N (10.44%).

*2-((2-(2-(4-chlorobenzamido)benzoyl)hydrazono)methyl)benzoic acid* (**6l**) Yield (87%); white powder; m.p. 251–253 °C; IR (KBr) ν_max_: 3368–3278 (2 NH and OH), 1758 (CO), 1680, 1656 (2 C=O), 1579 (C=N) cm^−1^; ^1^H-NMR (DMSO-*d*_6_) δ: 12.37 (s, 1H, NHCO), 11.95 (s, 1H, NHCO), 8.74 (s, 1H, N=CH), 8.52 (d, 1H, Ar-H, *J* = 8.3 Hz), 8.09 (d, 1H, Ar-H, *J* = 7.8 Hz) 7.97 (d, 2H, 2 × Ar-H, *J* = 8.5 Hz), 7.95–7.62 (m, 6H, 6 × Ar-H), 7.56 (t, 1H, Ar-H, *J* = 7.4 Hz), 7.28 (t, 1H, Ar-H, *J* = 7.4 Hz); ^13^C-NMR (DMSO-*d*_6_) δ: 168.51 (COOH), 165.61 (CO), 164.02 (CO), 148.38 (N=CH), 139.56, 137.39, 134.82, 133.66, 133.09, 132.49, 131.29, 130.35, 129.47, 129.25, 127.23, 123.78, 121.70, 121.19 (18 × Ar-C). MS (ESI): 423 [M^+^ + 1], Anal. Calcd. for C_22_H_16_ClN_3_O_4_: C (62.64%); H (3.82%); N (9.96%); Found: C (62.67%); H (3.85%); N (9.88%).

*4-Chloro-N-(2-(2-(3-hydroxy-4-methoxybenzylidene)hydrazine-1-carbonyl)phenyl)benzamide* (**6m**) Yield (94%); white powder; m.p. 239–241 °C; IR (KBr) ν_max_: 3383–3256 (2 NH), 1680, 1657 (2 C=O), 1582 (C=N) cm^−1^; ^1^H-NMR (DMSO-*d*_6_) δ: 12.04 (s, 1H, NHCO), 11.97 (s, 1H, NHCO), 8.56 (d, 1H, Ar-H, *J* = 8.3 Hz), 8.34 (s, 1H, N=CH), 7.96 (d, 2H, 2 × Ar-H, *J* = 8.5 Hz), 7.90 (d, 1H, *J* = 7.7 Hz), 7.67 (d, 2H, 2 × Ar-H, *J* = 8.5 Hz), 7.62 (t, 1H, Ar-H, *J* = 7.5 Hz), 7.31 (d, 1H, Ar-H, *J* = 1.6 Hz), 7.28 (t, 1H, Ar-H, *J* = 7.6 Hz), 7.08 (dd, 1H, Ar-H, *J* = 8.3, 1.6 Hz), 6.98 (d, 1H, Ar-H, *J* = 8.3 Hz), 3.82 (s, 3H, O-CH_3_); ^13^C-NMR (DMSO-*d*_6_) δ: 165.16 (CO), 163.93 (CO), 150.53, (C-OCH_3_), 149.81 (C-OH), 147.37 (N=CH), 139.58, 137.44, 133.62, 132.62, 129.49, 129.43, 129.01, 127.29, 123.77, 121.55, 121.13, 112.90, 112.29 (16 × Ar-C), 56.04 (OCH_3_). MS (ESI): 425 [M^+^ + 1], Anal. Calcd. for C_22_H_18_ClN_3_O_4_: C (62.34%); H (4.28%); N 00(9.91%); Found: C (62.38%); H (4.26%); N (9.93%).

*4-Chloro-N-(2-(2-(4-cyclohexylbenzylidene)hydrazine-1-carbonyl)phenyl)benzamide* (**6n**) Yield (85%); white crystals; m.p. 257–259 °C; IR (KBr) ν_max_: 3386–3260 (2 NH), 1682, 1654 (2 C=O), 1589 (C=N) cm^−1^; ^1^H-NMR (DMSO-*d*_6_) δ: 12.03 (s, 1H, NHCO), 11.08 (s, 1H, NHCO), 8.51 (d, 1H, Ar-H, *J* = 8.2 Hz), 8.36 (s, 1H, N=CH), 7.95 (d, 2H, 2 × Ar-H, *J* = 8.5 Hz), 7.88 (d, 1H, Ar-H, *J* = 7.7 Hz), 7.65 (d, 2H, 2 × Ar-H, *J* = 8.3 Hz), 7.59 (t, 1H, Ar-H, *J* = 7. 7Hz), 7.31–7.18 (m, 5H, 5 × Ar-H), 3.09–1.55 (m, 11H, cyclohexyl-H); ^13^C-NMR (DMSO-*d*_6_) δ: 165.54 (CO), 163.86 (CO), 146.14 (N=CH), 139.23, 137.44, 133.55, 132.61, 129.47, 129.39, 128.84, 127.16, 126.60, 123.76, 121.72, 121.49 (18 × Ar-C), 42.85, 41.23, 35.32, 34.39, 33.43, 28.39 (cyclohexyl-C). MS (ESI): 461 [M^+^ + 1], Anal. Calcd. For C_27_H_26_ClN_3_O_2_: C (70.50%); H (5.70%); N (9.14%); Found: C (70.51%); H (5.71%); N (9.13%).

*(Z)-4-chloro-N-(2-(2-(3-hydroxybenzylidene)hydrazine-1-carbonyl)phenyl)benzamide* (**6o**) Yield (95%); white powder; m.p. 262–264 °C; IR (KBr) ν_max_: 3386–3258 (2 NH, OH), 1680, 1652 (2 C=O), 1579 (C=N) cm^−1^; ^1^H-NMR (DMSO-*d*_6_) δ: 12.12 (s, 1H, NHCO), 12.04 (s, 1H, NHCO), 8.84 (s, 1H, N=CH), 8.55 (d, 1H, Ar-H, *J* = 8.3 Hz), 7.97 (d, 2H, 2 × Ar-H, *J* = 8.5 Hz), 7.95–7.90 (m, 2H, 2 × Ar-H), 7.67 (d, 2H, 2 × Ar-H, *J* = 8.5 Hz), 7.63 (t, 1H, Ar-H, *J* = 7.5 Hz), 7.46–7.03 (m, 4H, 4 × Ar-H); ^13^C-NMR (DMSO-*d*_6_) δ: 165.27 (CO), 163.96 (CO), 158.40 (C-OCH_3_), 145.10 (N=CH), 139.64, 137.42, 133.64, 133.05, 132.40, 129.50, 129.46, 129.09, 126.09, 123.77, 122.47, 121.48, 121.57, 121.23, 120.99 (17 × Ar-C). MS (ESI): 395 [M^+^ + 1], Anal. Calcd. For C_21_H_16_ClN_3_O_3_: C (64.05%); H (4.09%); N (10.67%); Found: C (64.07%); H (4.12%); N (10.63%).

### 3.2. Biological Evalustion

#### 3.2.1. Inhibition of sPLA_2_ Activity

The test of inhibitory activity of secretory phospholipase A_2_ (sPLA_2_) was performed as described by De Aranjo and Radvany [50]. Briefly, the substrate consisted of 3.5 mM lecithin in a mixture of 3 mM NaTDC, 100 mM NaCl, 10 mM CaCl_2_ and 0.055 mM red phenol as colorimetric indicator in 100 mL H_2_O. The pH of the reaction mixture was adjusted to 7.6 using phosphate buffer. The Human group IIA (hG-IIA) and dromedary group IB (DrG-IIA) sPLA_2_ were solubilized in 10% acetonitrile at a concentration of 0.05 μg/μL. A volume of 10 μL of these PLA_2_ solutions was incubated with 10 μL containing 10 μg of each compound for 20 min at room temperature. Then, 1 mL of the PLA_2_ substrate was added, and the kinetic of hydrolysis was followed during 5 min by reading the optical density at 558 nm. The inhibition percentage was calculated by comparison with a control experiment (absence of compound).

#### 3.2.2. Protease Inhibitor Assay

Three commercially available proteases; proteinase K (Sigma-Aldrich, St. Louis, MO, USA, P2308), esperase (Novozyme, Sigma-Aldrich, P5860) and that obtained from *Bacillus* sp. (Sigma-Aldrich, P3111) were evaluated for the effect of the studied compounds on their activities. Protease assays were carried out by adopting Kunitz caseinolytic method [51] using Hammerstein casein as substrate. Respective protease inhibitor activities were assayed under the same conditions with the addition of the inhibitor (0.1 mg/mL) to the respective reaction mixture and pre incubation for 10 min at 37 °C. The assay of the residual enzyme activity was followed by the addition of 2 mL of 1% casein and the resulting mixture was allowed to stand for 30 min at 37 °C. The reaction was stopped by the addition of 2.5 mL of 5% TCA solution. After centrifugation of the reaction mixture (12,000 rpm, 15 min), the absorbance was measured at 280 nm. Protease inhibitor unit is defined as the amount of protease inhibitor that inhibited one unit of respective enzyme activity. The protease inhibitor activity was expressed in terms of percent inhibition. Appropriate blanks for the enzyme, inhibitor, and the substrate were also included in the assay along with the test.

#### 3.2.3. Antibacterial Activity

##### Culture of Microbial Strains Preparation

Pure standard microbial isolates collected from King Khaled University Hospital were tested in this study; including *Staphylococcus aureus* ATCC 25923, Methicillin resistant *Staphylococus aureus* ATCC 12498, *Bacillus subtilis* ATCC 6633, *Enterococcus faecalis* ATCC 29212 as Gram-positive and *Escherichia coli* ATCC 25966, *Pseudomonas aeruginosa* ATCC 27853 and *Serratia marcescens* as Gram-negative bacteria. Fresh cultures of each microorganism were grown on nutrient agar plates (Oxoid, Thermo Scientific, Basingstoke, UK); of which small inoculums were suspended in 5 mL nutrient broth for bacterial suspension preparation of 0.5 MacFarland.

##### Antibacterial Assay

Antimicrobial activity was assayed using well diffusion technique according to given literature [43]. Briefly, small inoculums of each of the microbial suspension prepared were loaded on sterile Muller Hinton agar plates surface (Oxoid) with sterile cotton swabs. Loaded plates were then perforated equidistantly with a sterile 6 mm diameter cork borer, and 70 μL of each compound solution (1 mg/mL) were loaded in their respective wells. DMSO and Tetracycline were used as negative and positive controls, respectively. Plates were kept to rest for 30 min at room temperature and then incubated at 37 °C for 18–24 h. Antimicrobial activity was determined by measuring the inhibition zone. All tests were performed in duplicates and means of inhibition zones were recorded in mm.

### 3.3. Molecular Docking

In the present work, AutoDock 4.0 (The Scripps Research Institute, La Jolla, CA, USA) was used for molecular docking. The software is freely available and can be used with different molecular viewers like PMV, PyMol, etc. For docking simulations, the structures of the molecules were drawn using ChemSketch 12.0 (Advanced Chemistry Development, Inc., Toronto, ON, Canada) freeware and optimized using the inbuilt methodology. The optimized structures were saved in 3D-format in .mol file format. The structures of proteins GIIA sPLA_2_ (pdb: 1DB4), proteinase K (pdb: 2PWB) and glutamine-fructose-6-phosphate transaminase (1JXA) were retrieved from www.rcsb.org. The pdb 1DB4, 2PWB, and 1JXA for the proteins were selected on the basis of reasonable X-ray resolution and sequence completion. The proteins structures were optimized before actual docking simulations. Following parameters were set for getting better docking results: Algorithm: genetic Algorithm; Number of runs: 10 GA runs; Number of evaluations: 250,000 evaluation/run; population size: 150.

## 4. Conclusions

In conclusion, fifteen novel hydrazone derivatives **6a**–**o** were synthesized, fully characterized and examined to evaluate their inhibitory activity against two phospholipases A_2_, three protease enzymes and a panel of Gram-negative and Gram-positive bacterial strains. Among the investigated compounds; **6e** and **6l** selectively exhibited sPLA_2_ the highest inhibitory activity against *h*G-IIA isoform. It is quite interesting to report that compound **6l** also displayed the highest antiprotease inhibition against all used protease enzymes.

Moreover, the series under investigation showed varied antibacterial inhibitory activity. Thus, while similar inhibition to that produced by the reference drug tetracycline was displayed by compound **6a** against *P. aeruginosa*, compounds **6m** and **6o** exhibited higher inhibition. In addition, the most susceptible bacterial strain to the synthesized compounds was genus *Serratia*, which was inhibited by nine of them, with compound **6a** being the most powerful inhibitory agent. Furthermore, the maximum inhibitory activities were exhibited by compounds **6j**, **6l**, and **6n** against *S. aureus* and by compound **6k** against *S. mutans*. Finally, compounds **6a**, **6d**, **6e**, **6m** and **6n** were found to be more potent than the used reference drug against *E. feacalis*. The docking simulations of compounds **6a**–**o** with GIIA sPLA_2_, proteinase K and compounds **6a**–**e** GlcN6P were carried out in order to investigate the mode of action.

## Figures and Tables

**Table 1:** Antibacterial activities of the synthesized hydrazones **6a**–**o**.

Comp #	*Gram-Negative Bacteria*					*Gram-Positive Bacteria*					
	*E. coli*	*p. aeruginosa*	*K. Pneumoniae*	*Salmonella*	*Serratia*	*S. aureus*	*S. aureus ALA1*	*MRSA ATCC3*	*S. mutans*	*E. feacalis*	*B. subtilis*
**6a**	0 ± 0	17 ± 1	0 ± 0	8 ± 1	15.5 ± 0	0 ± 0	10 ± 0	12 ± 0	12 ± 0.5	18.5 ± 0.5	0 ± 0
**6b**	14.5 ± 1.5	12 ± 0	0 ± 0	12.5 ± 0.5	12.5 ± 0.5	8 ± 0.5	0 ± 0	6 ± 0	0 ± 0	9.5 ± 0.5	0 ± 0
**6c**	1 ± 0	1.5 ± 0	0 ± 0	10.25 ± 1	1.4 ± 1	0 ± 0	0 ± 0	8 ± 0.5	0.6 ± 1.2	1.3 ± 1	0 ± 0
**6d**	14.5 ± 0.5	10 ± 0	12.2 ± 1	6.5 ± 0	12 ± 0	8.5 ± 0.5	0 ± 0	0 ± 0	10 ± 0.0	18 ± 1	0 ± 0
**6e**	10 ± 0	12.5 ± 0.1	12.5 ± 0.5	4 ± 0	14 ± 1	8.5 ± 0.5	0 ± 0	14 ± 1	8 ± 1	18.5 ± 0.5	0 ± 0
**6f**	0 ± 0	10.5 ± 0.1	4.5 ± 0.1	10 ± 0	14 ± 0.1	0 ± 0	0 ± 0	0 ± 0	8 ± 1	0 ± 0	0 ± 0
**6g**	0 ± 0	10.5 ± 0.5	8 ± 1	0 ± 0	10.5 ± 0.5	8.5 ± 0	0 ± 0	0 ± 0	0 ± 0	0 ± 0	0 ± 0
**6h**	8 ± 0	12.5 ± 0.7	0 ± 0	0 ± 0	10 ± 0	8.5 ± 0.7	0 ± 0	0 ± 0	0 ± 0	0 ± 0	0 ± 0
**6i**	8.5 ± 0.5	10.5 ± 0.5	0 ± 0	0 ± 0	8.5 ± 0.5	10.5 ± 1.5	0 ± 0	0 ± 0	0 ± 0	0 ± 0	0 ± 0
**6j**	8 ± 1	10 ± 0	0 ± 0	0 ± 0	8.5 ± 0.5	19.5 ± 1.5	0 ± 0	0 ± 0	0 ± 0	0 ± 0	0 ± 0
**6k**	10 ± 0	13.5 ± 1.5	14.5 ± 1.5	0 ± 0	10 ± 0	10.5 ± 1.5	0 ± 0	0 ± 0	24.5 ± 0.5	0 ± 0	8.5 ± 0.5
**6l**	14 ± 1	11 ± 0	11 ± 0	8 ± 1	13 ± 0.1	18.5 ± 0.5	0 ± 0	0 ± 0	14 ± 2	0 ± 0	0 ± 0
**6m**	10 ± 1	18.5 ± 0.5	0 ± 0	4 ± 0	14.5 ± 1.5	8.5 ± 0.5	0 ± 0	8.5 ± 0.5	0 ± 0	13.5 ± 1.5	0 ± 0
**6n**	10 ± 0	0 ± 0	13 ± 1	0 ± 0	12.5 ± 0.5	14 ± 1	0 ± 0	10 ± 0	15 ± 1	18.5 ± 0.5	0 ± 0
**6o**	10 ± 0	18.5 ± 0.5	10 ± 0	0 ± 0	12.5 ± 0.5	0 ± 0	0 ± 0	0 ± 0	9 ± 0.6	0 ± 0	0 ± 0
TCN	19 ± 0.1	17 ± 0.2	17 ± 0.5	16 ± 0.1	12 ± 0.3	31 ± 0.6	29 ± 0.4	18 ± 0.3	27 ± 1.2	0 ± 0	15 ± 0.5

Comp # = Compound number; TCN = Tetracycline.

## Supplementary Materials

Supplementary materials can be accessed at: http://www.mdpi.com/1420-3049/21/12/1664/s1.
