# Supplementary material for: Synthesis, Antiphospholipase A2, Antiprotease, Antibacterial Evaluation and Molecular Docking Analysis of Certain Novel Hydrazones
Source: Molecules. 2016 Dec 2;21(12):1664. doi: 10.3390/molecules21121664 (PMC6272960; doi:10.3390/molecules21121664)
Supplement: Supplementary file 1 [file molecules-21-01664-s001.pdf]

Supplementary Materials: Synthesis, Antiphospholipase A2, Antiprotease, Antibacterial Evaluation and Molecular Docking Analysis of Certain Novel Hydrazones

Nahed N. E. El-Sayed, Ahmed M. Alafeefy, Mohammed A. Bakht, Vijay H. Masand, Ali Aldalbahi, Nan Chen, Chunhai Fan and Abir Ben Bacha

Table S1. Inhibitory activity of compounds **6a-o** against sPLA2 (hG-IIA and DrG-IB).

Compound	sPLA2 Inhibitory Activity (%)	
	*h*G-IIA	DrG-IB
**6a**	39.67 ± 2.51	2.66 ± 2.51
**6b**	37.33 ± 4.04	11.67 ± 3.51
**6c**	50.67 ± 4.04	14.67 ± 2.51
**6d**	55.67 ± 4.72	10 ± 3.00
**6e**	72.67 ± 2.51	4.66 ± 0.57
**6f**	37.33 ± 3.51	6.33 ± 0.57
**6g**	10.67 ± 0.57	9 ± 1.73
**6h**	15.33 ± 2.51	8.33 ± 1.15
**6i**	26.67 ± 3.21	5.66 ± 1.52
**6j**	19.33 ± 2.08	6.66 ± 1.52
**6k**	35.33 ± 3.51	6.66 ± 2.08
**6l**	74.33 ± 3.21	11.33 ± 2.51
**6m**	17.67 ± 3.05	2.66 ± 1.15
**6n**	9 ± 2	4 ± 3.60
**6o**	12.67 ± 3.21	1.66 ± 1.15

Table S2. Antiprotease activity (%) of compounds **6a–o** against different protease enzymes.

Compound	Anti-Protease Activity (%)		
	Proteinase K	Protease from *Bacillus sp.*	Esperase
**6a**	27.33 ± 2.51	40.66 ± 4.04	6.66 ± 1.52
**6b**	55.66 ± 5.03	72.33 ± 2.51	13 ± 2.00
**6c**	34.66 ± 2.51	51 ± 3.60	15 ± 3.00
**6d**	26.33 ± 3.21	38 ± 2.64	20.66 ± 3.05
**6e**	74.66 ± 2.51	57 ± 4.00	31.33 ± 6.02
**6f**	39 ± 4.58	44 ± 1.00	12.33 ± 2.08
**6g**	57.33 ± 2.51	72.33 ± 6.42	24 ± 3.60
**6h**	14.66 ± 3.51	7.66 ± 2.51	5 ± 1.00
**6i**	30.66 ± 5.50	42 ± 6.55	14 ± 2.64
**6j**	35 ± 3.60	48.33 ± 3.51	17.66 ± 3.51
**6k**	50.66 ± 4.04	62.33 ± 2.51	24.33 ± 4.04
**6l**	86 ± 2.64	74.66 ± 2.88	39.33 ± 3.21
**6m**	69 ± 4.58	56.33 ± 3.51	32.33 ± 2.08
**6n**	44.66 ± 6.50	50 ± 2.64	21.33 ± 3.51
**6o**	26 ± 4.58	41 ± 3.00	34.33 ± 3.05

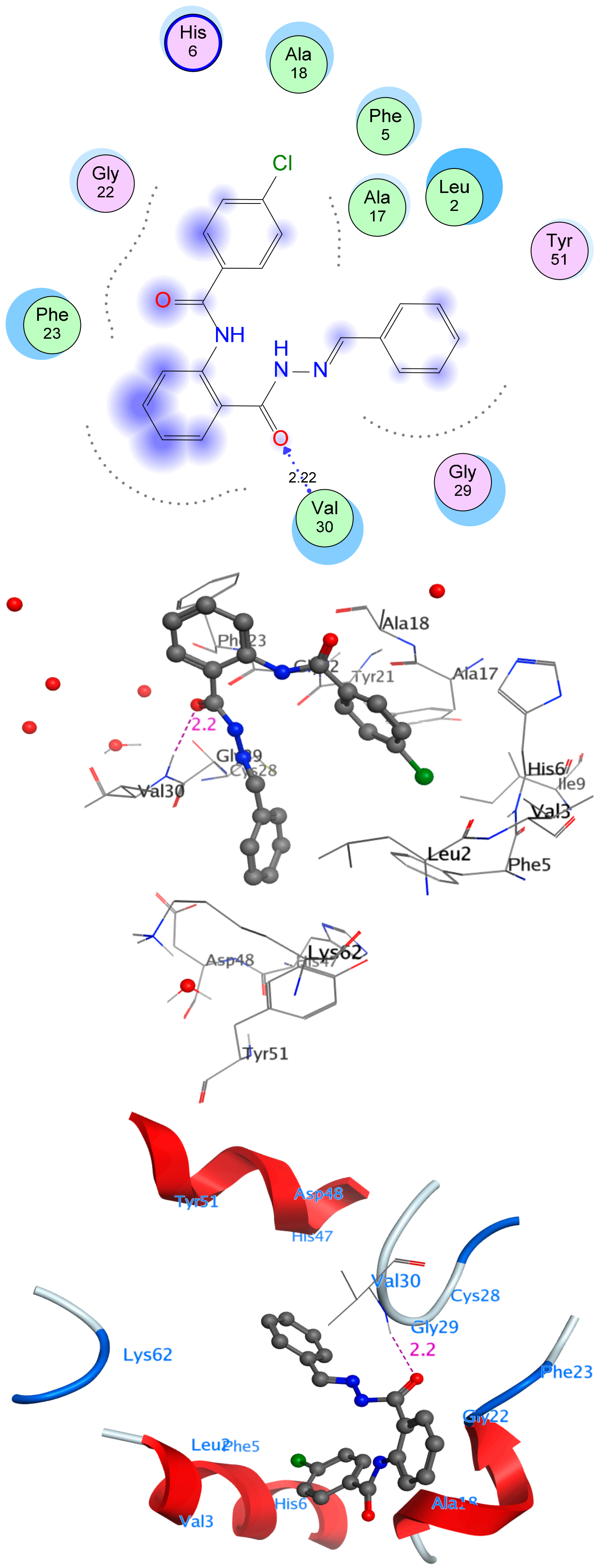

Docking Pose for Compound **6a** in the Active Site of GIIAsPLA2

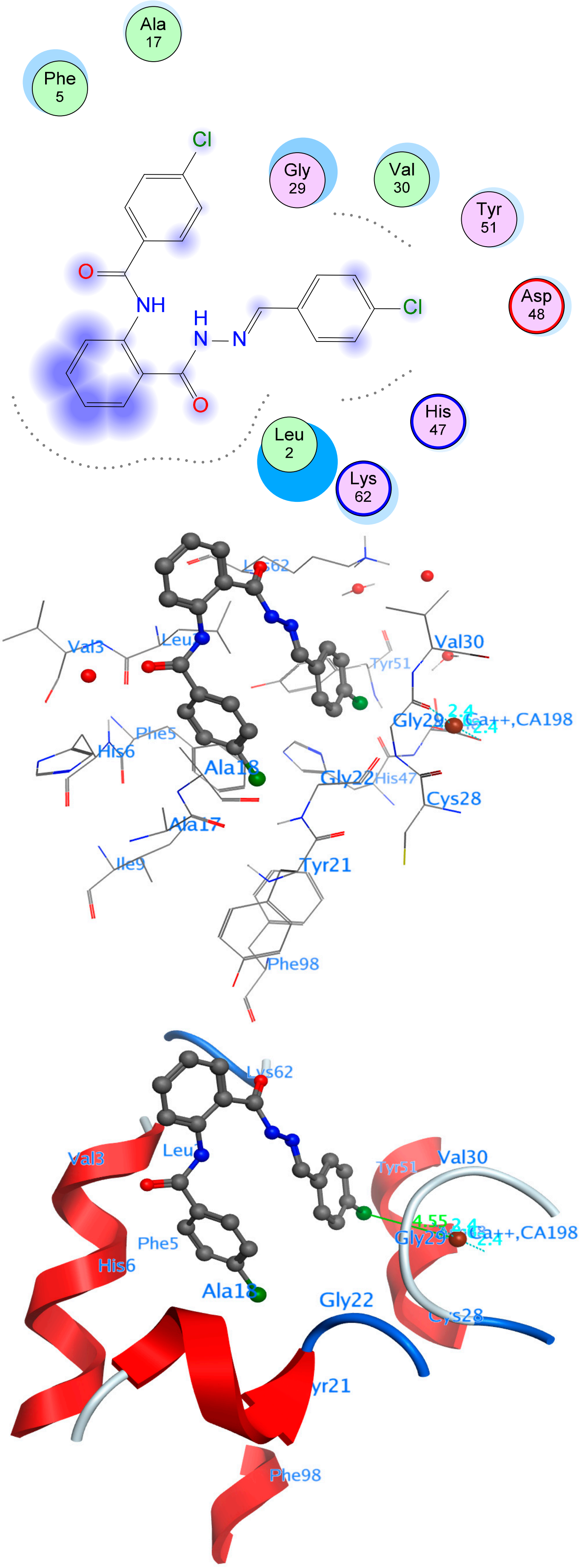

Docking pose for compound **6b** in the active site of GIIAsPLA2

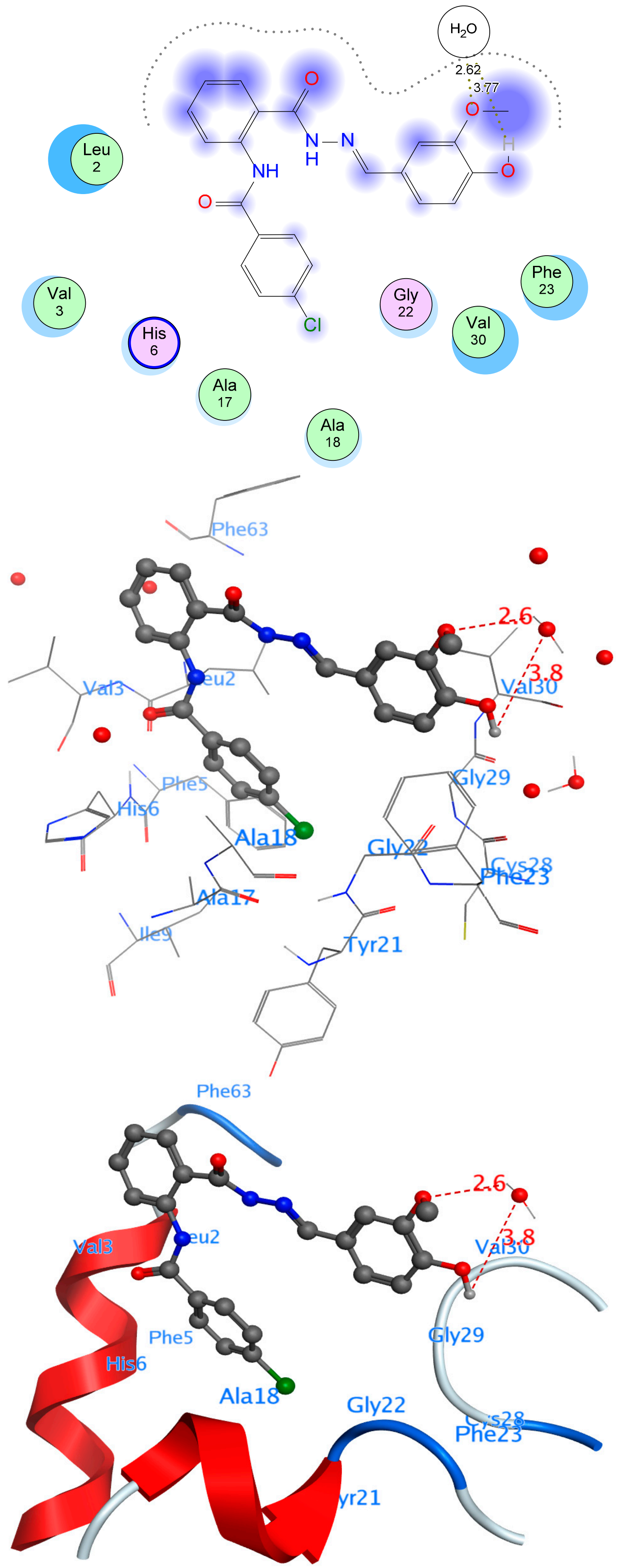

Docking pose for compound **6c** in the active site of GIIAsPLA2

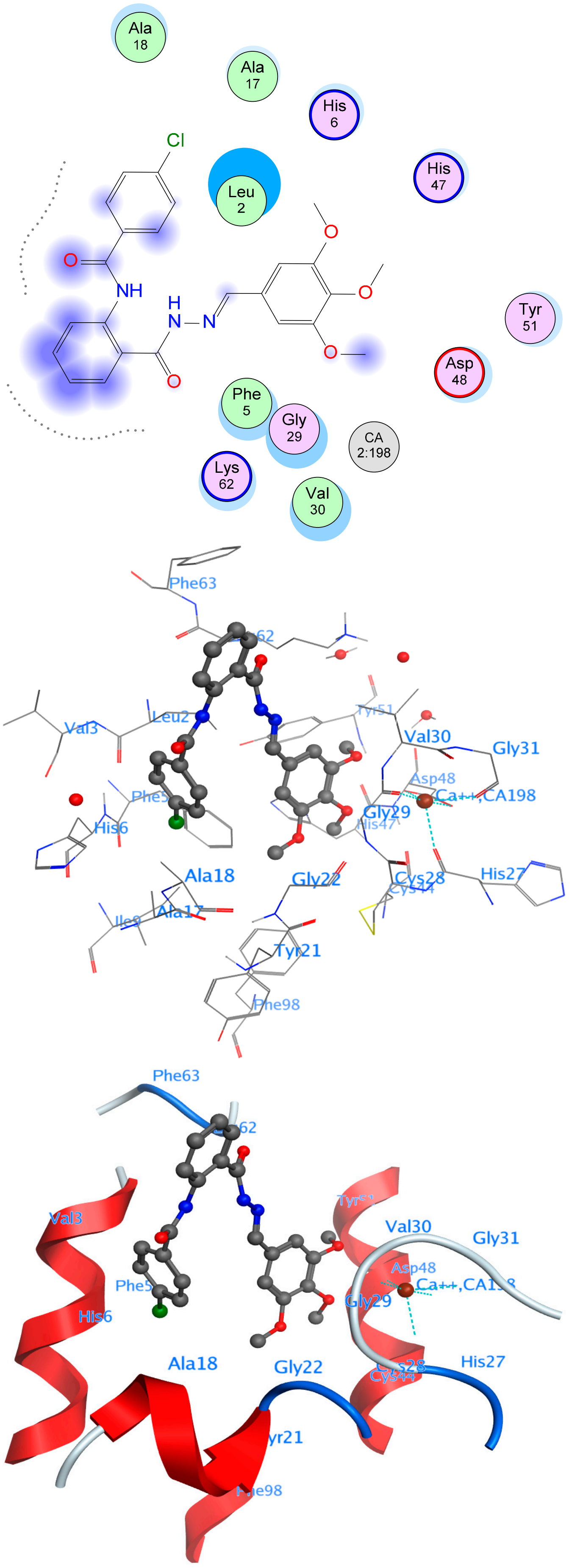

Docking pose for compound **6d** in the active site of GIIAsPLA2

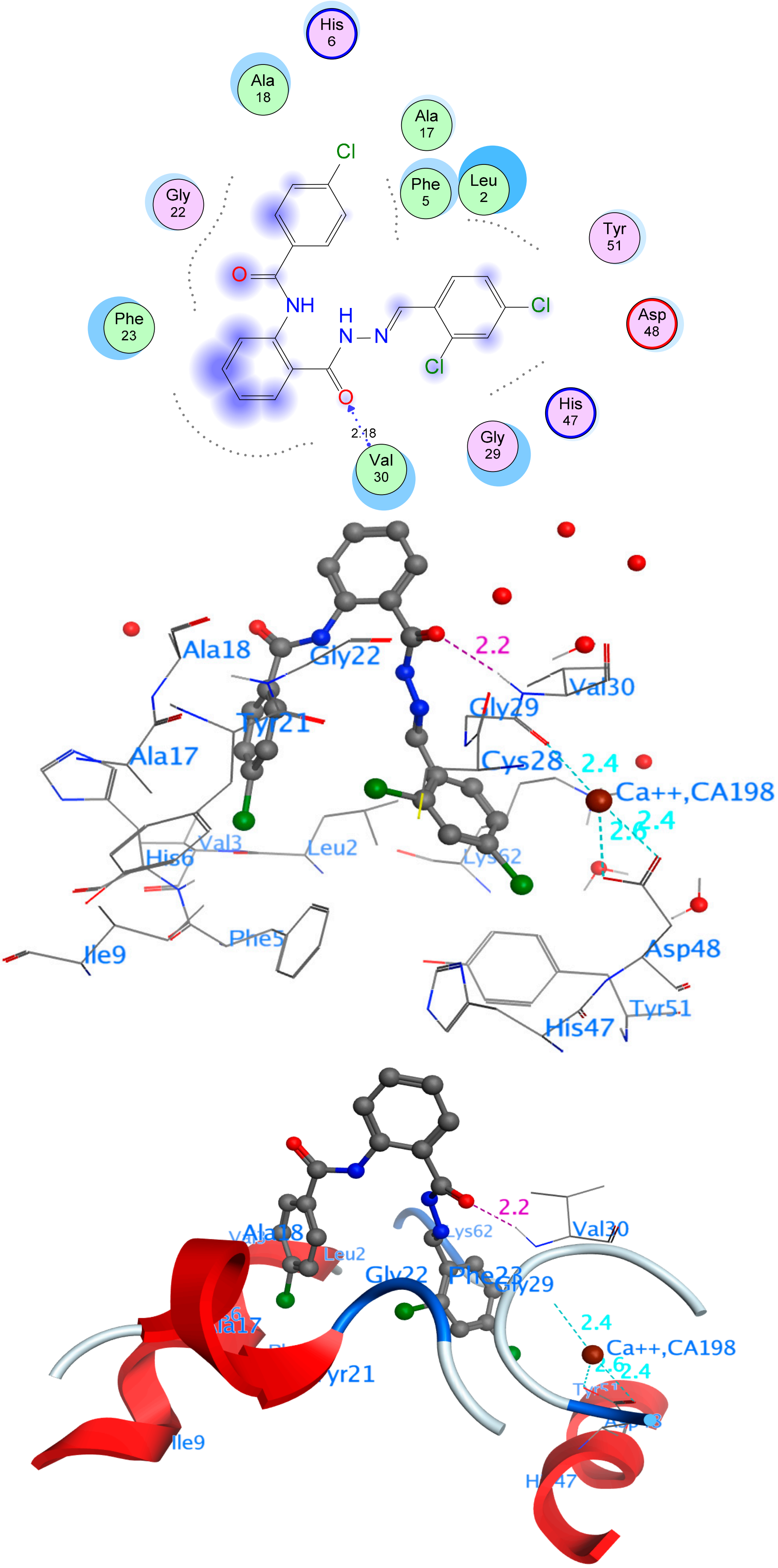

Docking pose for compound **6e** in the active site of GIIAsPLA2

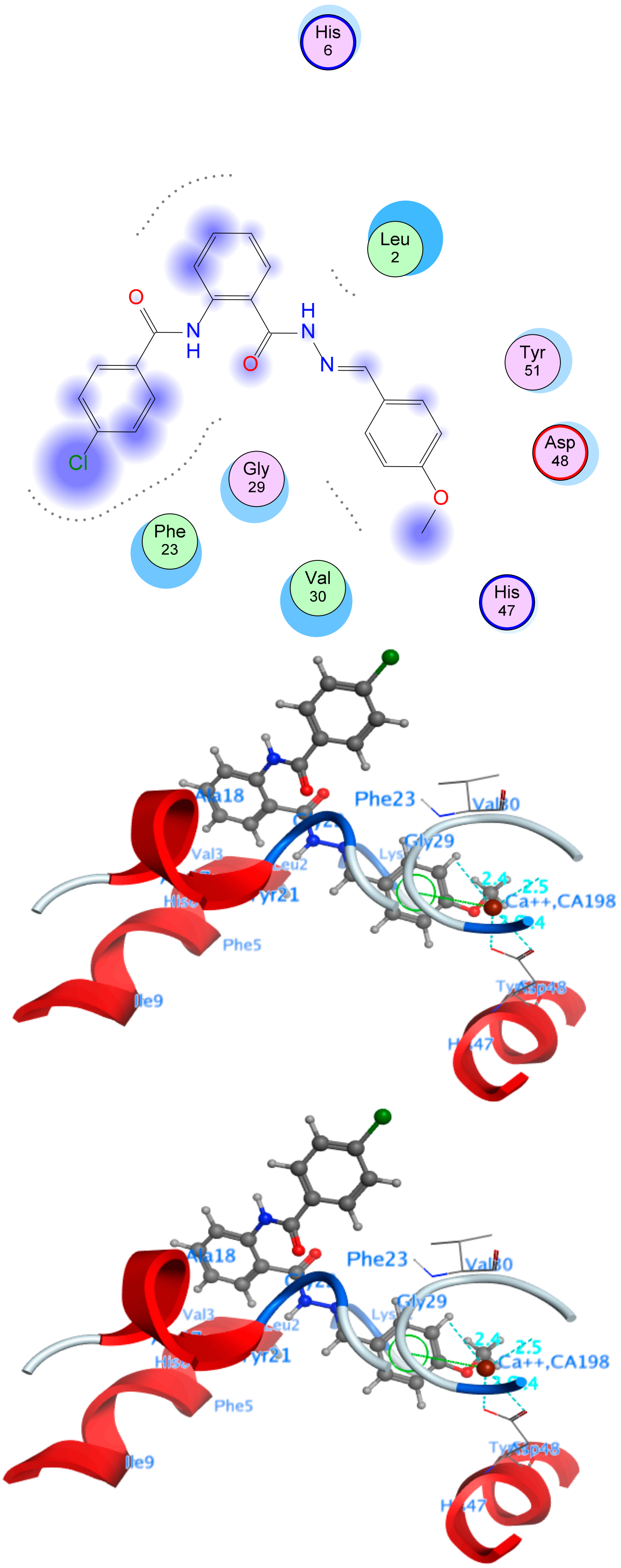

Docking pose for compound **6f** in the active site of GIIAsPLA2

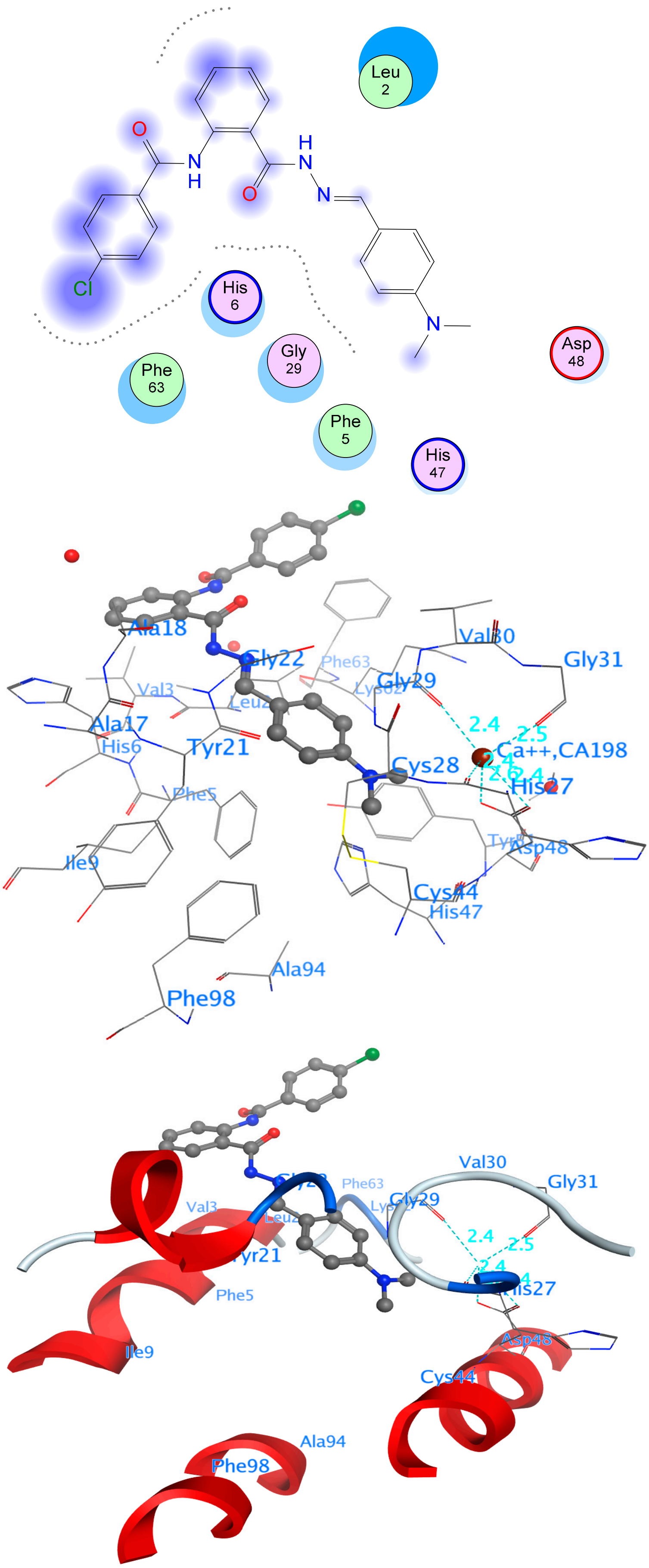

Docking pose for compound **6g** in the active site of GIIAsPLA$_2$

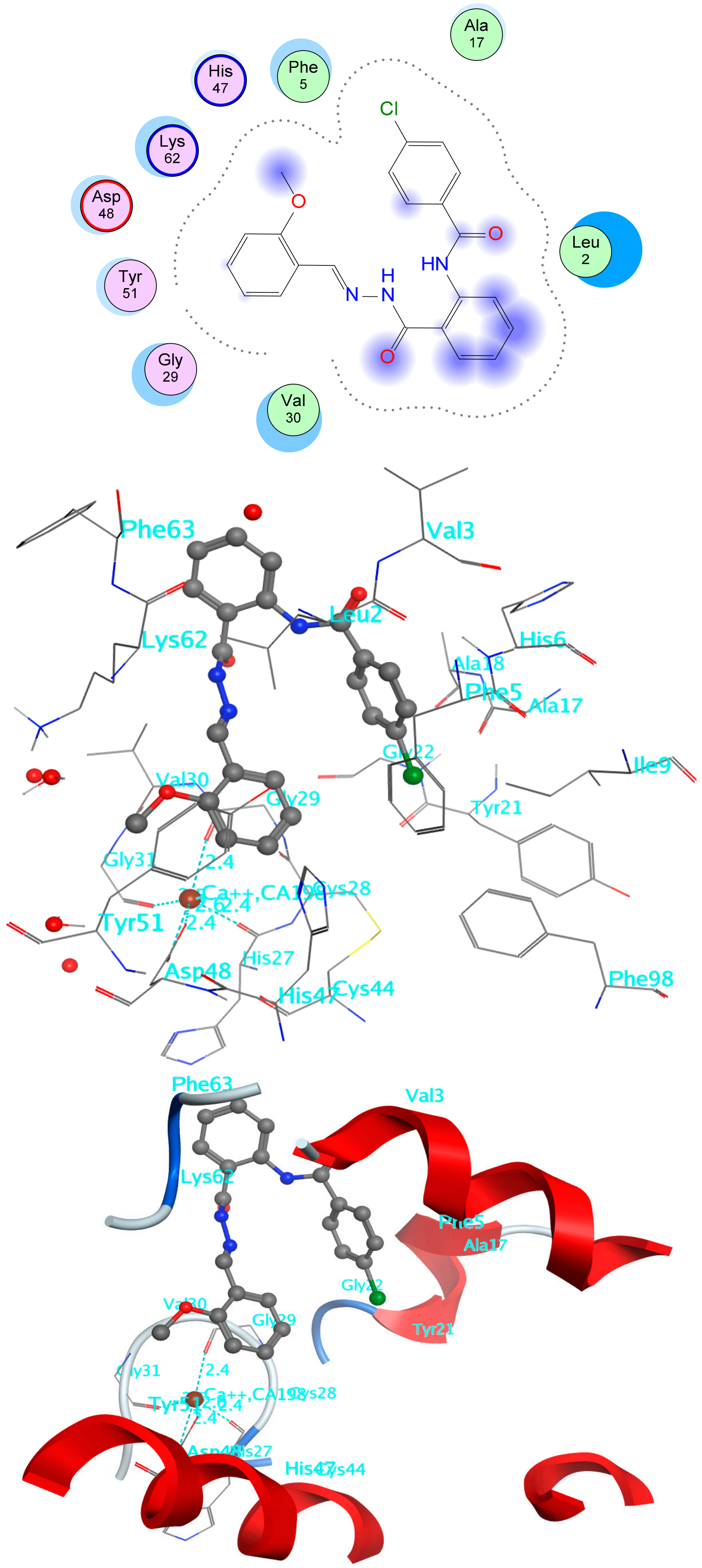

Docking pose for compound **6h** in the active site of GIIAsPLA2

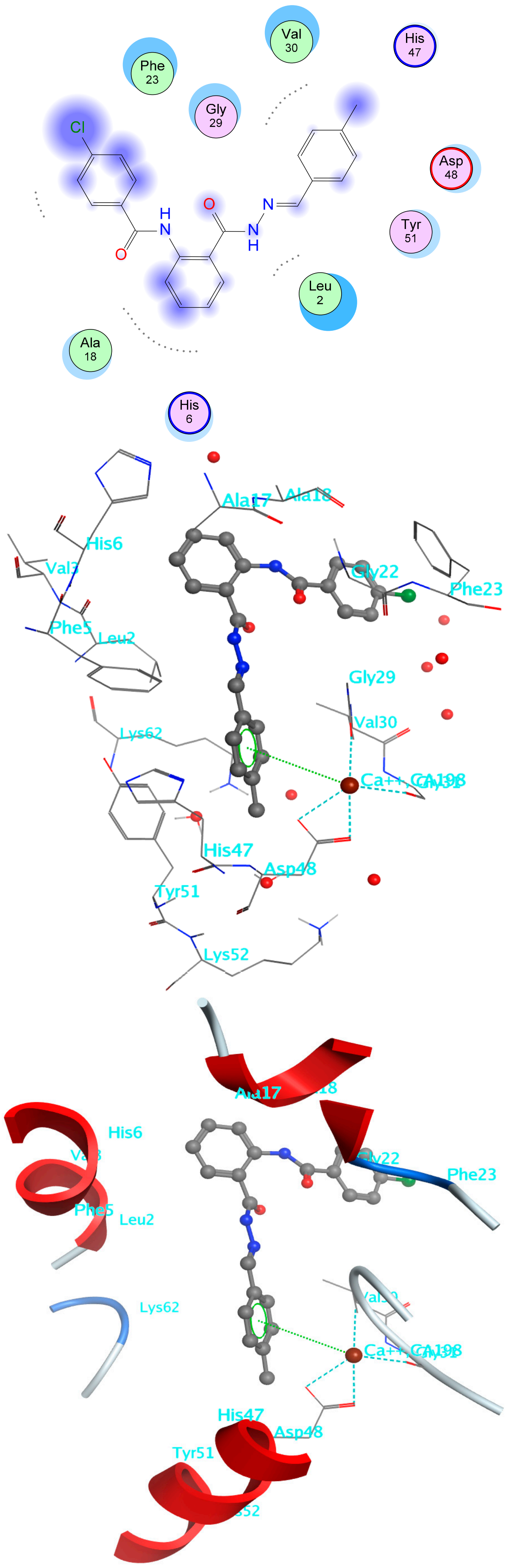

Docking pose for compound **6i** in the active site of $GIIAsPLA_2$

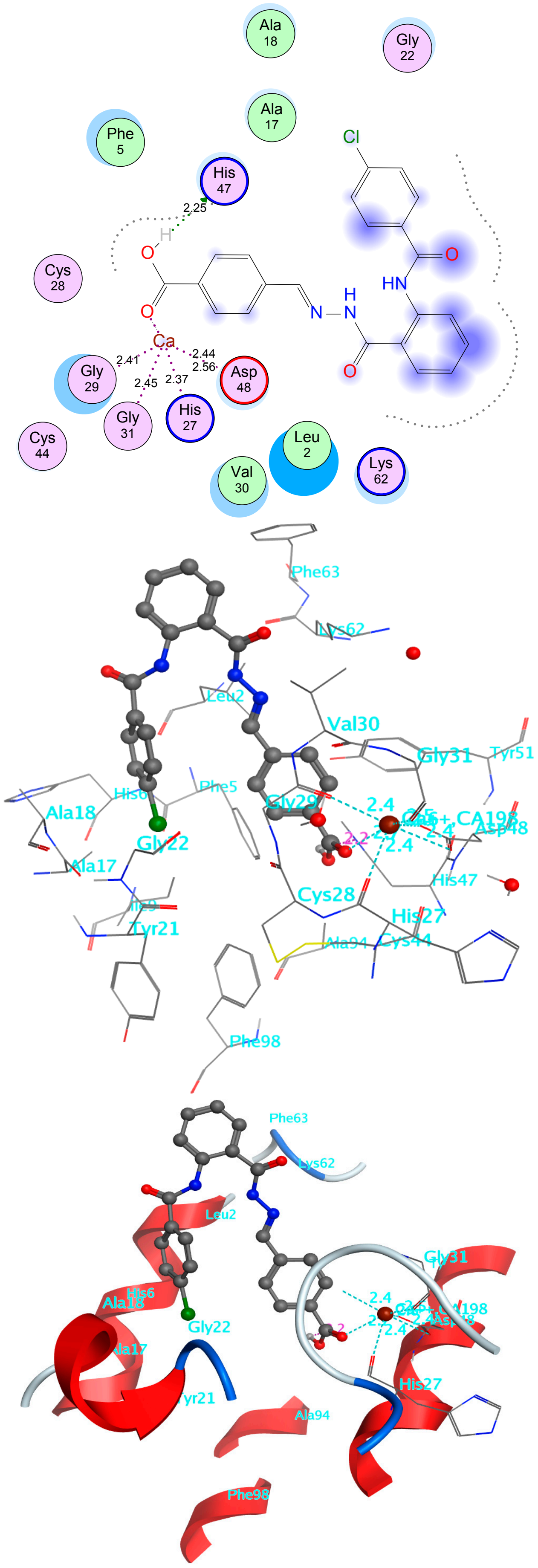

Docking pose for compound **6j** in the active site of GIIAsPLA_2

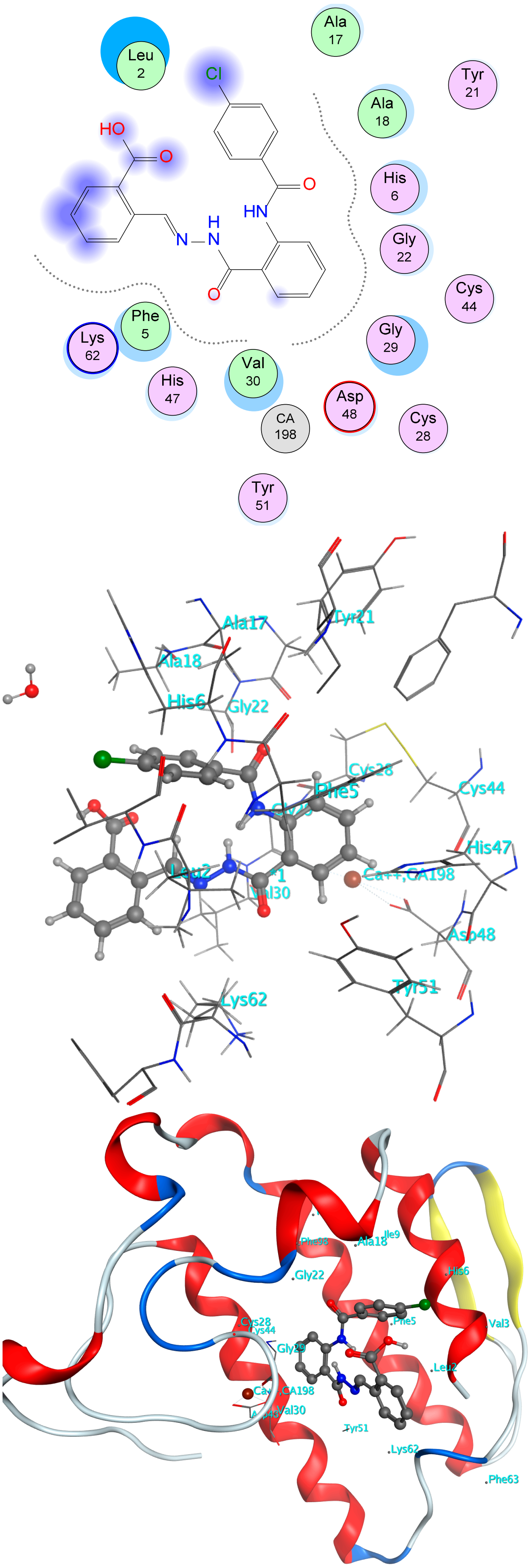

Docking pose for compound **6l** in the active site of GIIAsPLA$_2$

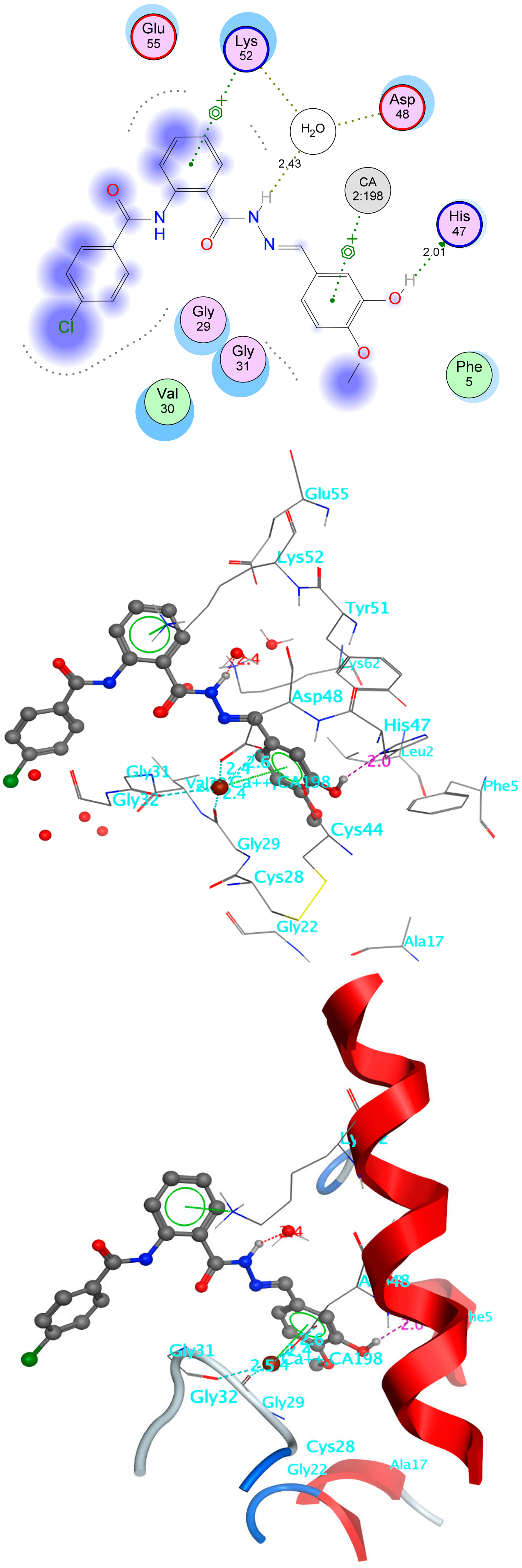

Docking pose for compound **6m** in the active site of GIIAsPLA2

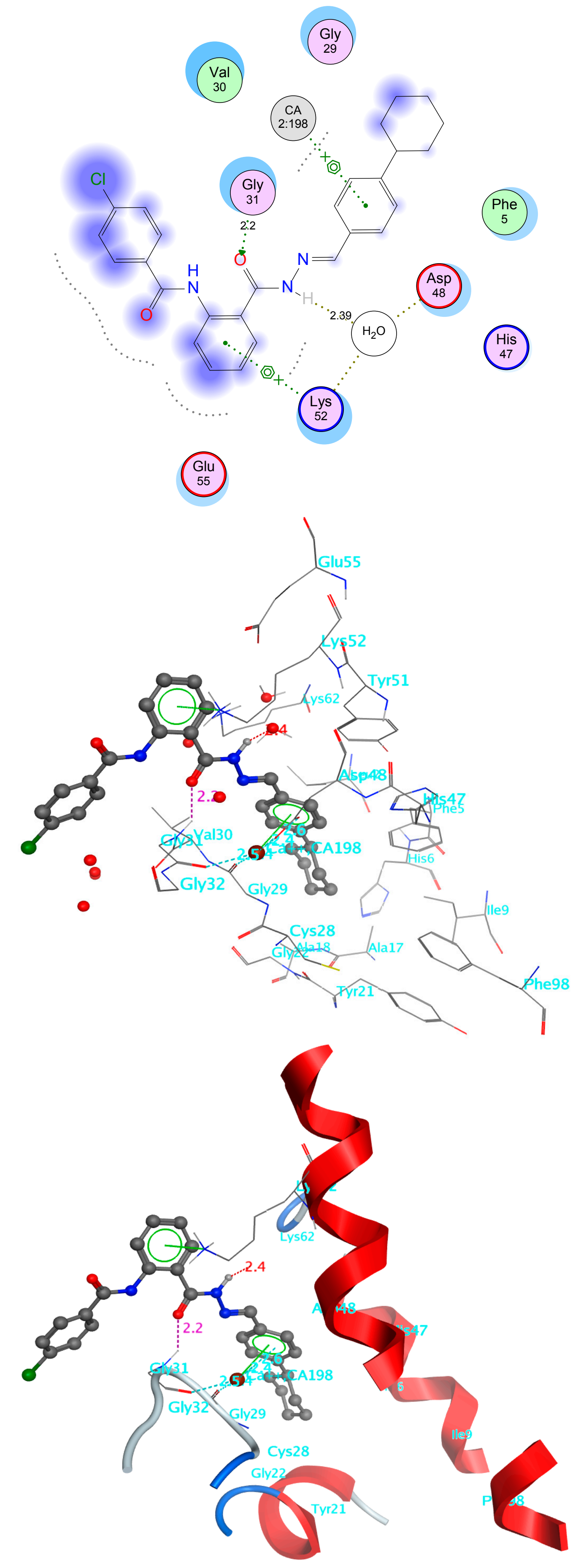

Docking pose for compound **6n** in the active site of GIIAsPLA2

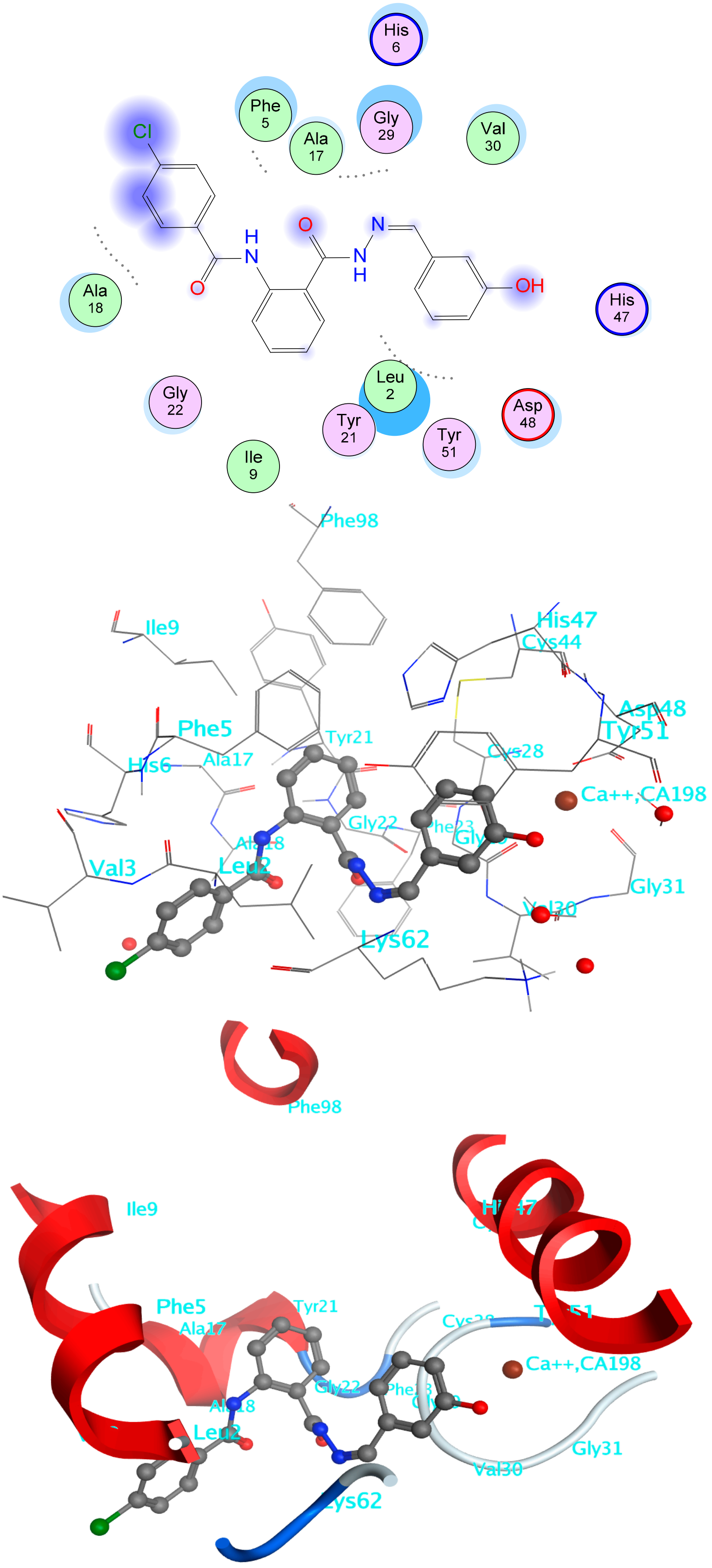

Docking pose for compound **6o** in the active site of GIIAsPLA$_2$

Figures S1. Docking poses of compounds **6a–o** in active site of GIIAsPLA$_2$.

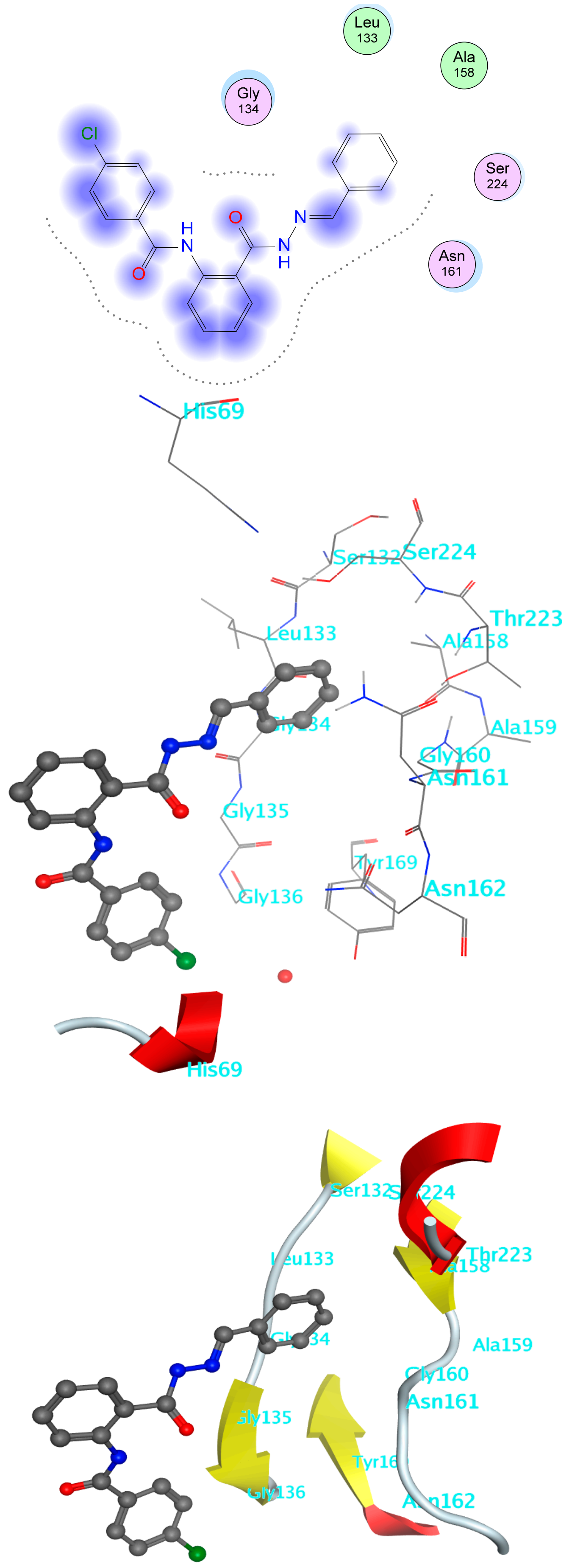

Docking pose for compound **6a** in the active site of Proteinase K

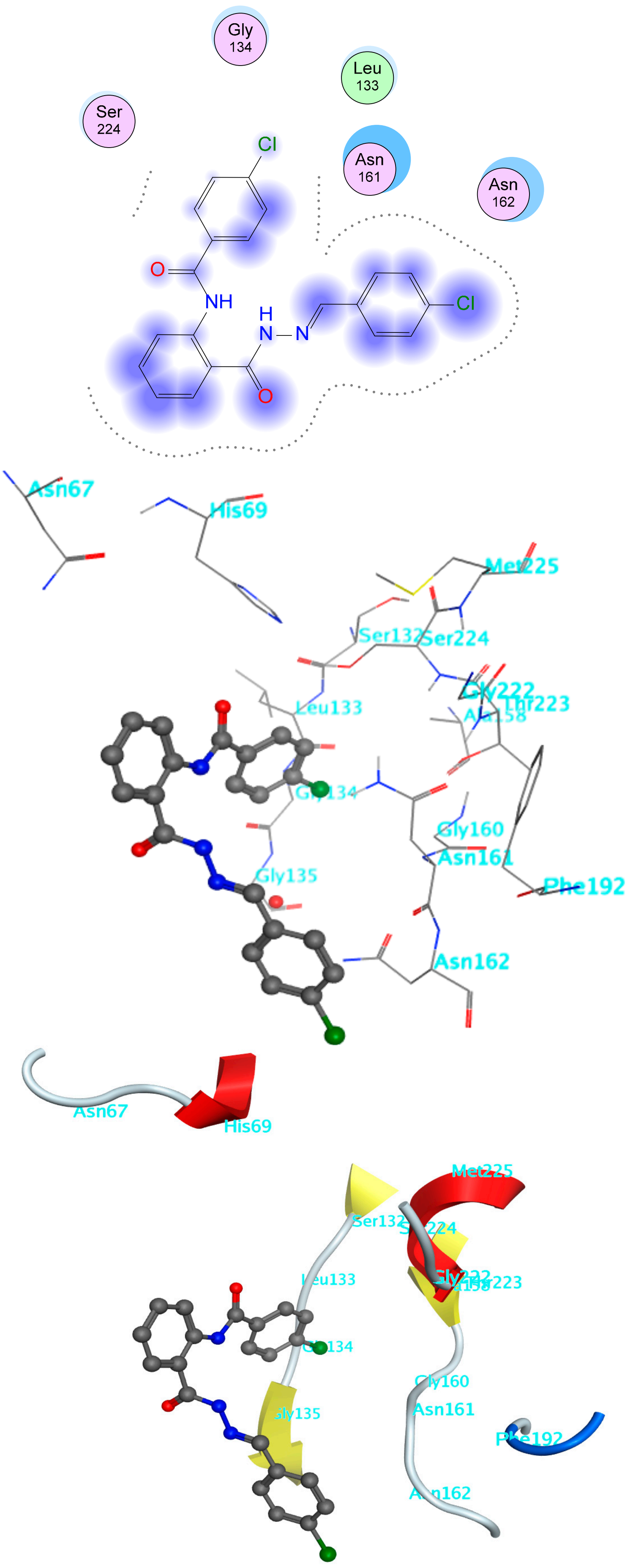

Docking pose for compound **6b** in the active site of Proteinase K

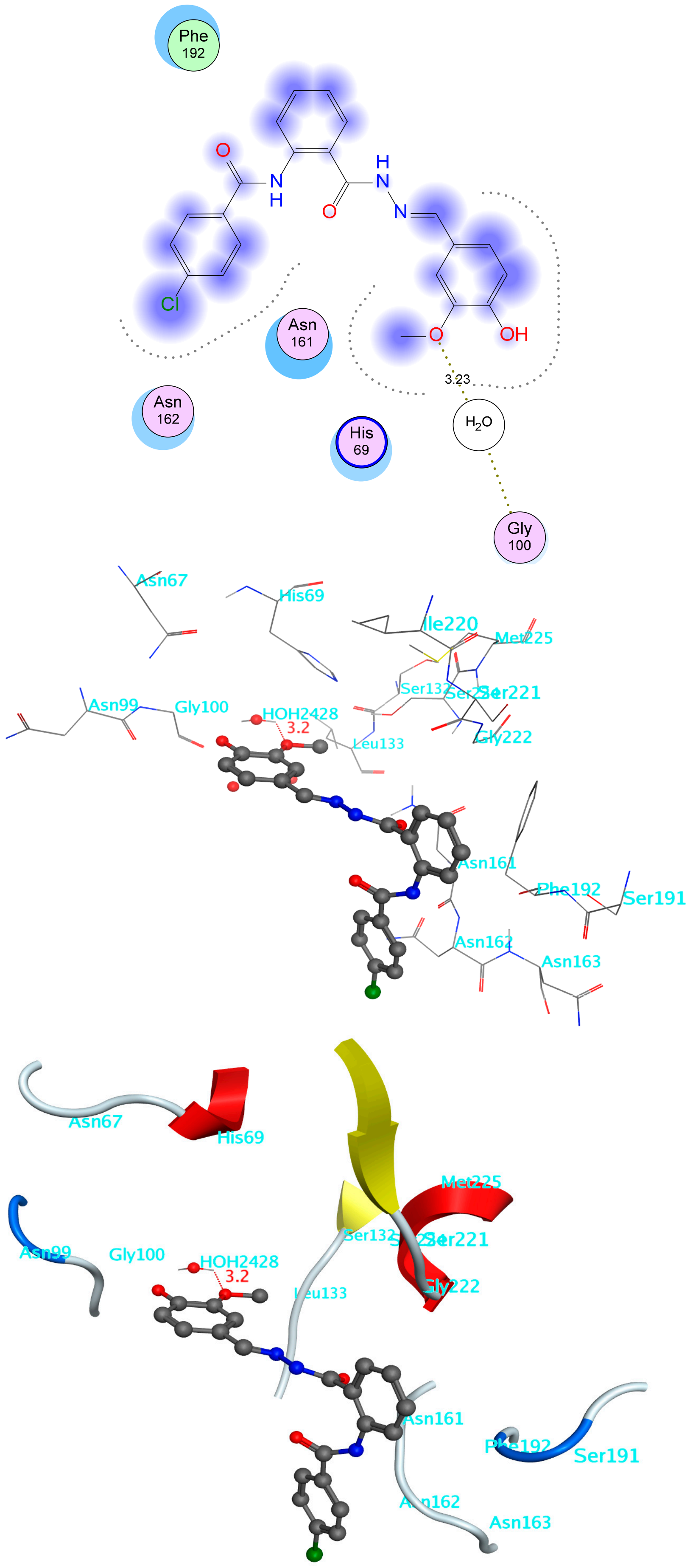

Docking pose for compound **6c** in the active site of Proteinase K

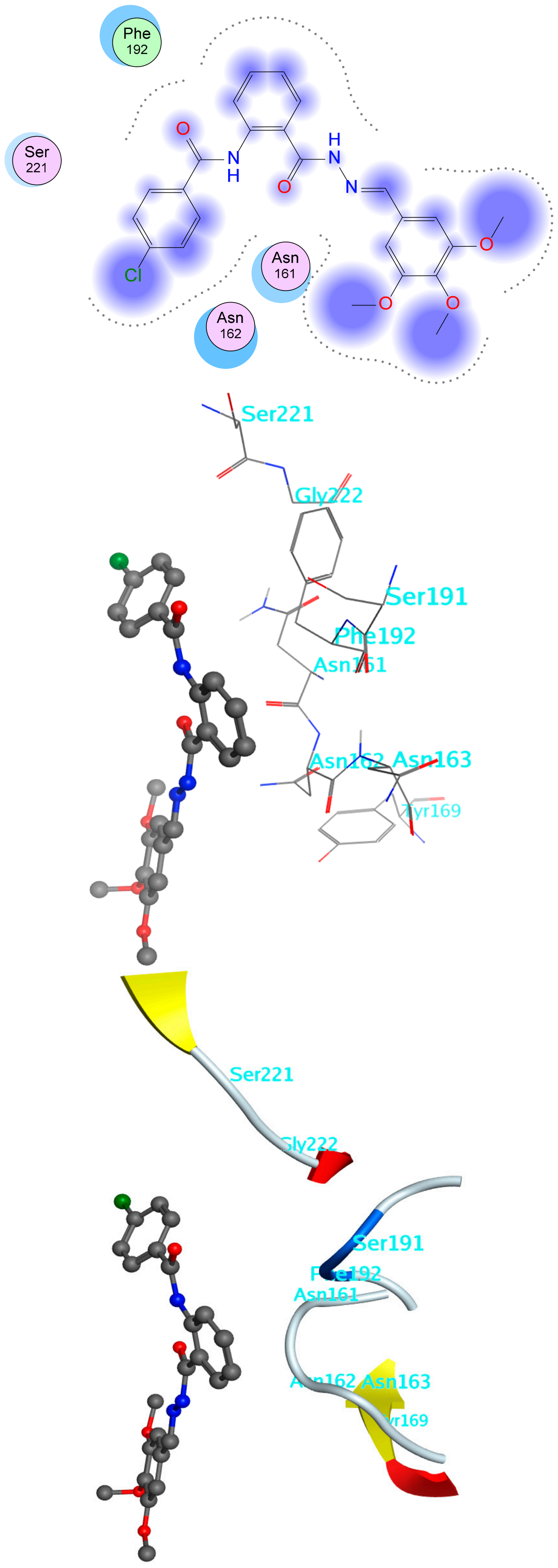

Docking pose for compound **6d** in the active site of Proteinase K

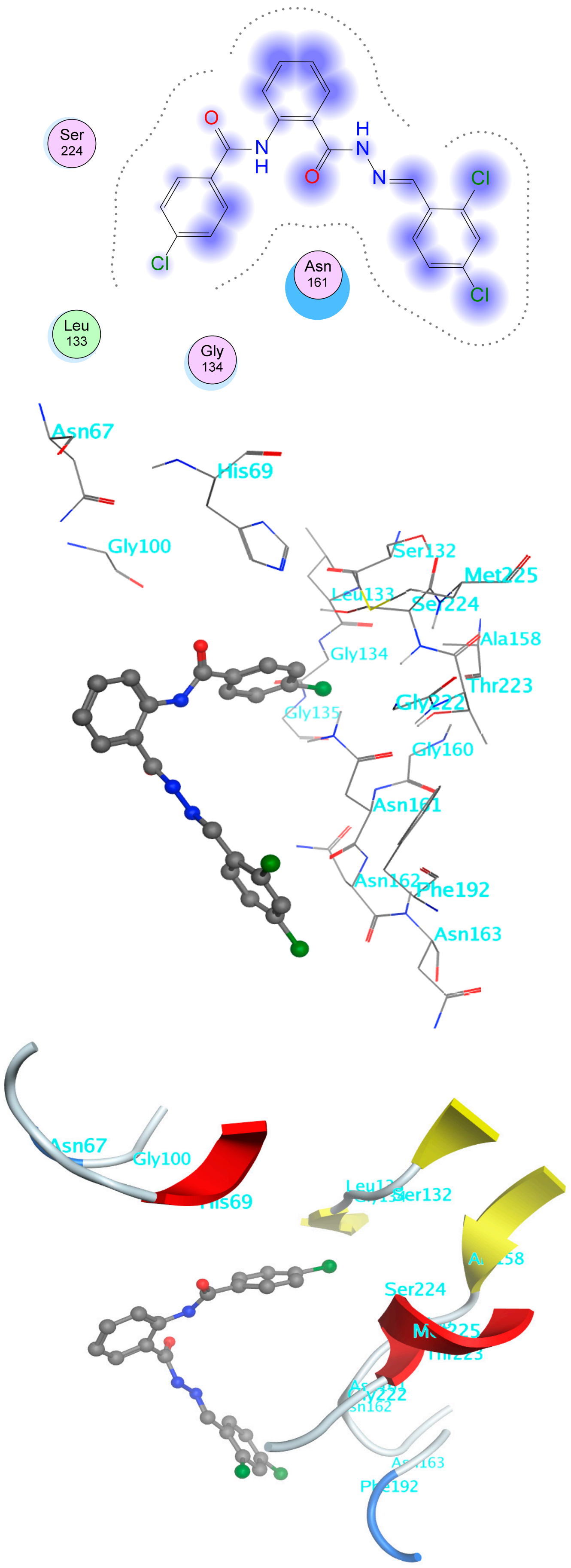

Docking pose for compound **6e** in the active site of Proteinase K

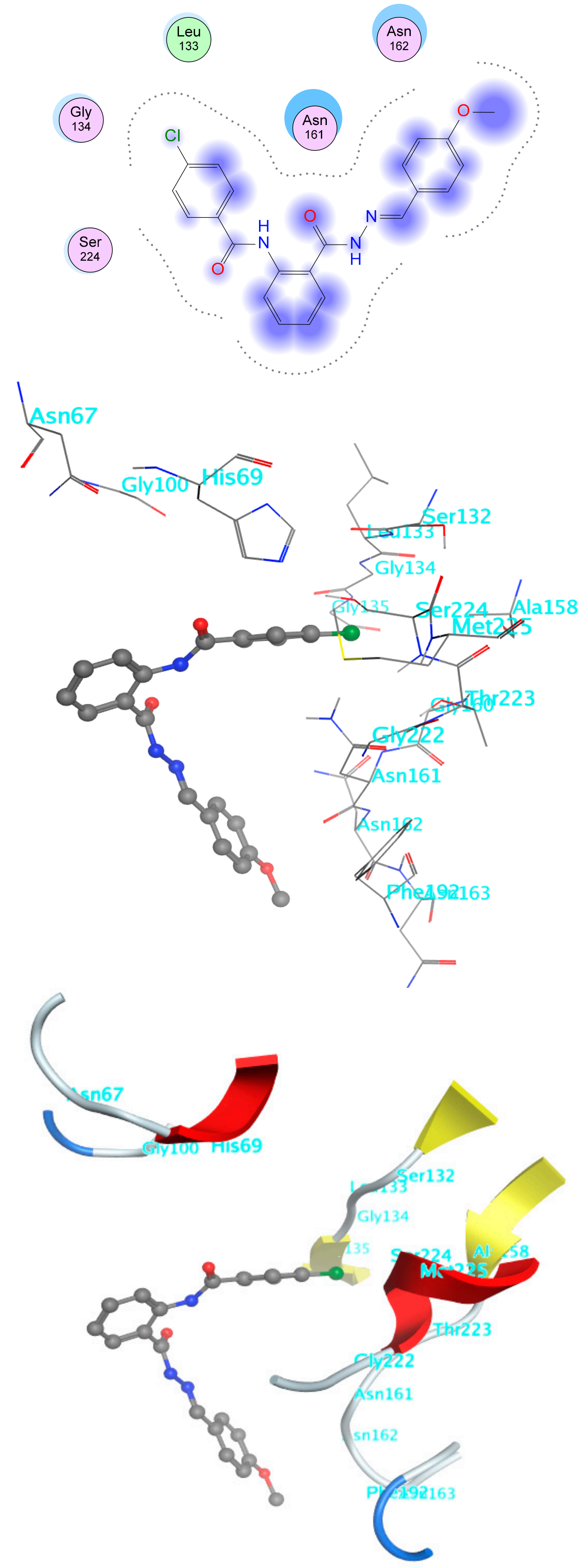

Docking pose for compound **6f** in the active site of Proteinase K

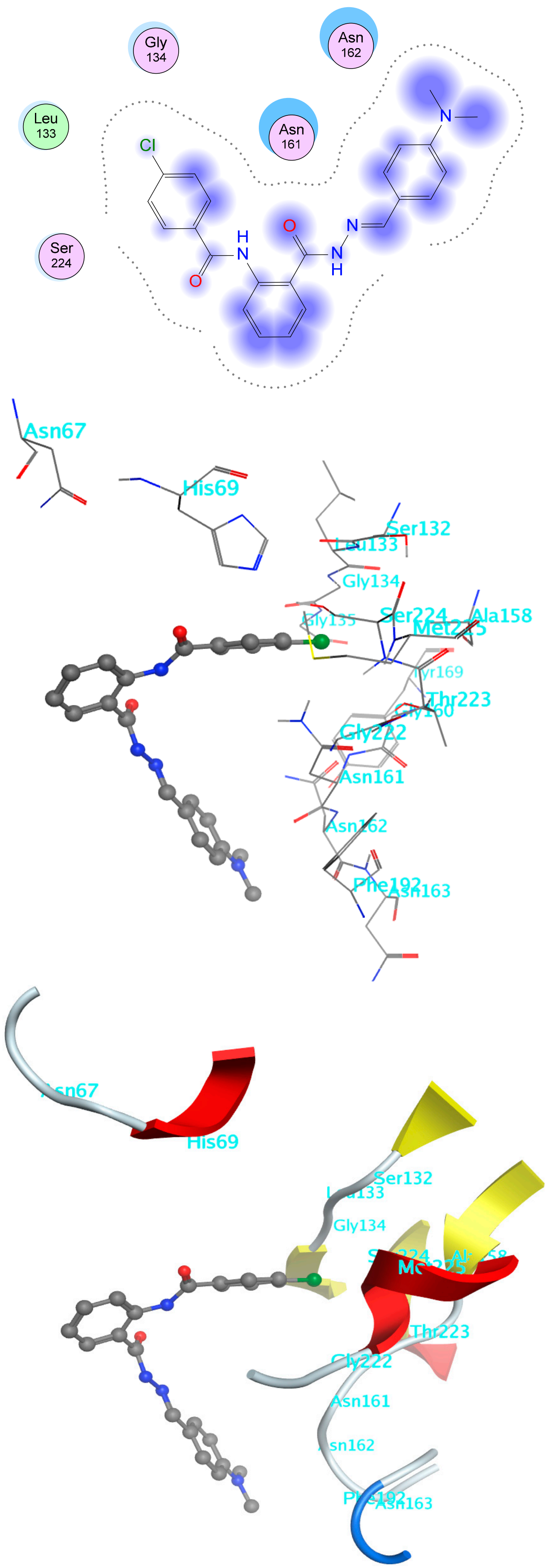

Docking pose for compound **6g** in the active site of Proteinase K

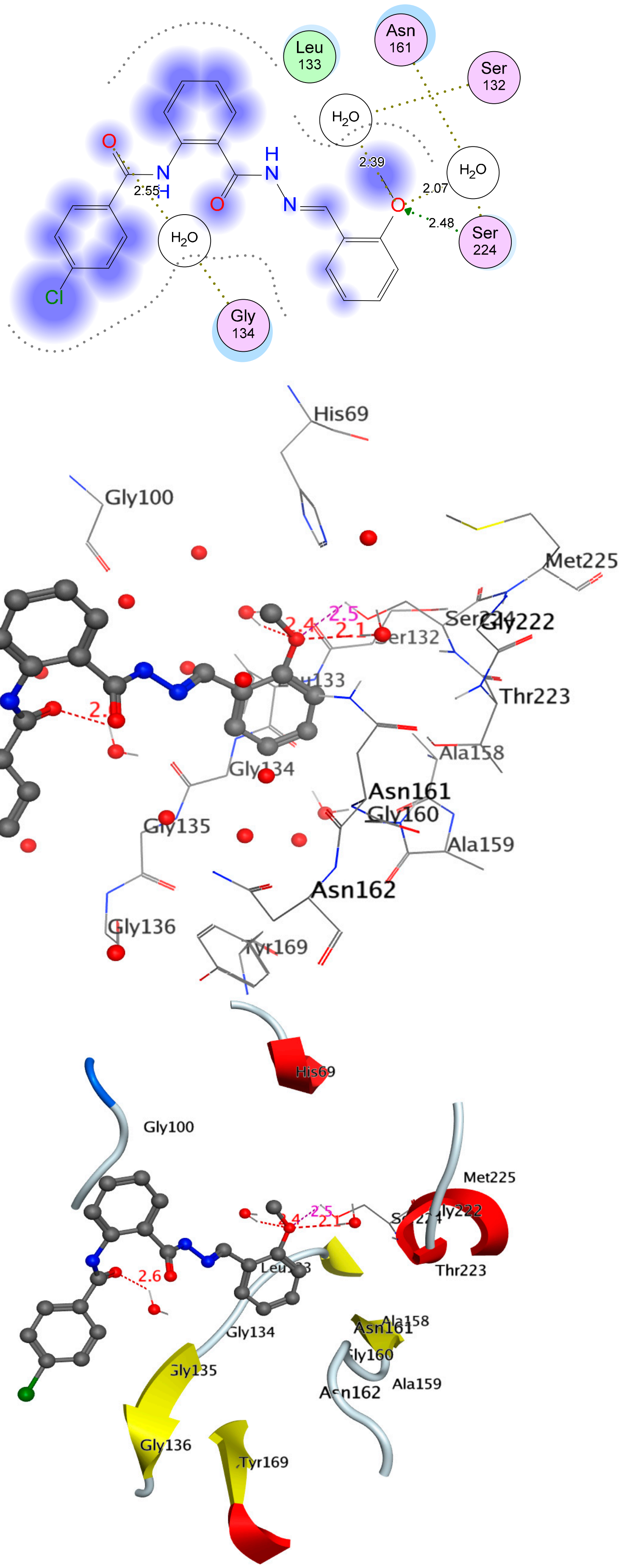

Docking pose for compound **6h** in the active site of Proteinase K

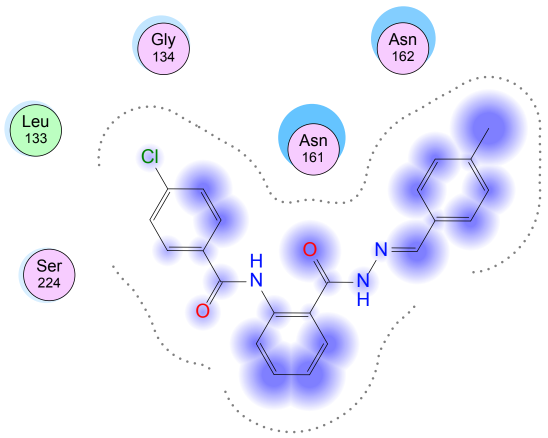

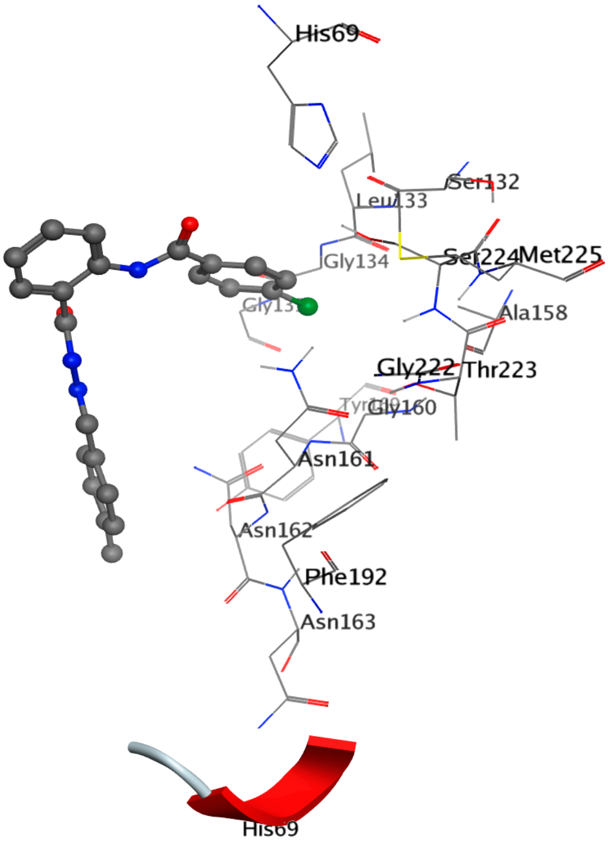

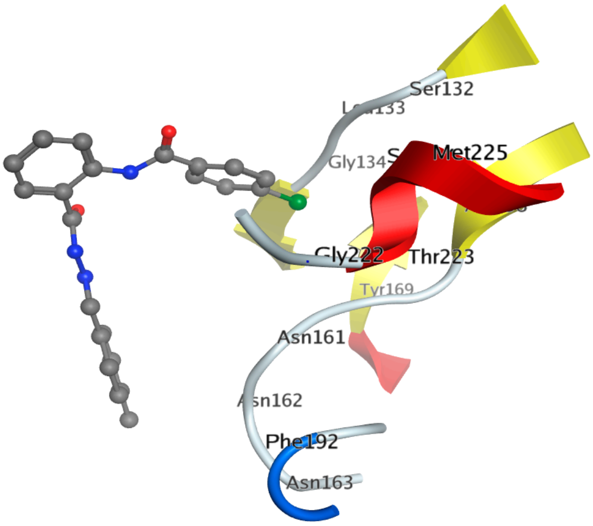

Docking pose for compound **6i** in the active site of Proteinase K

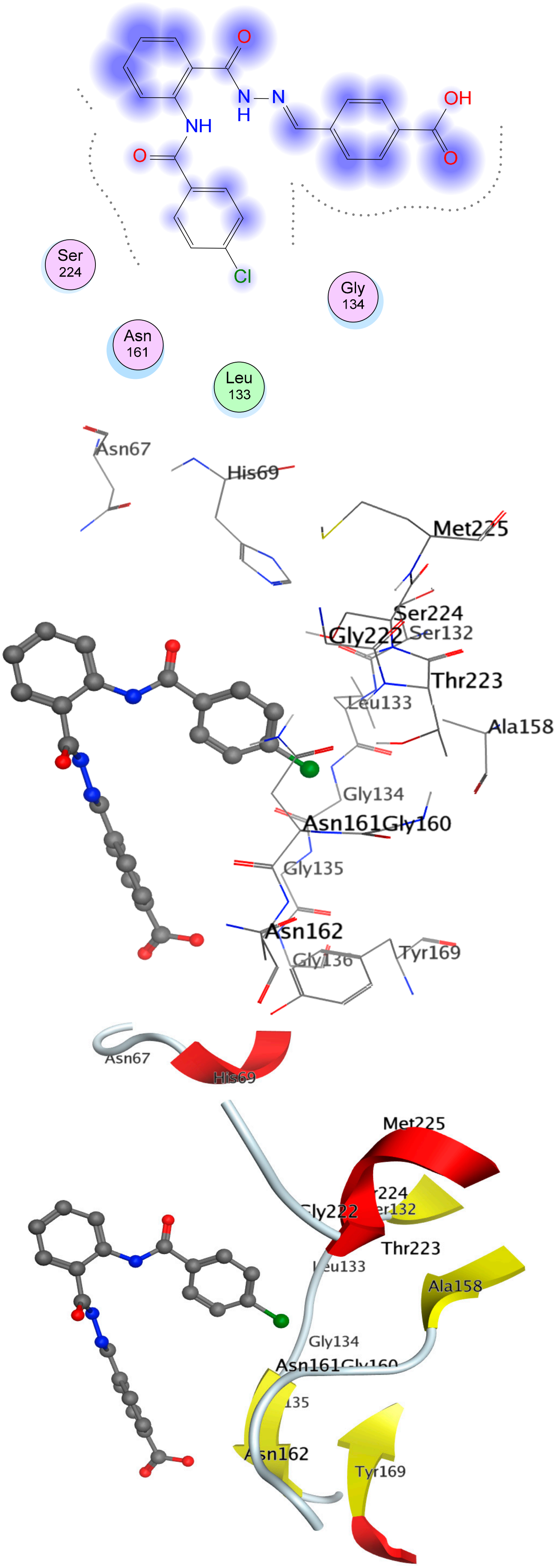

Docking pose for compound **6j** in the active site of Proteinase K

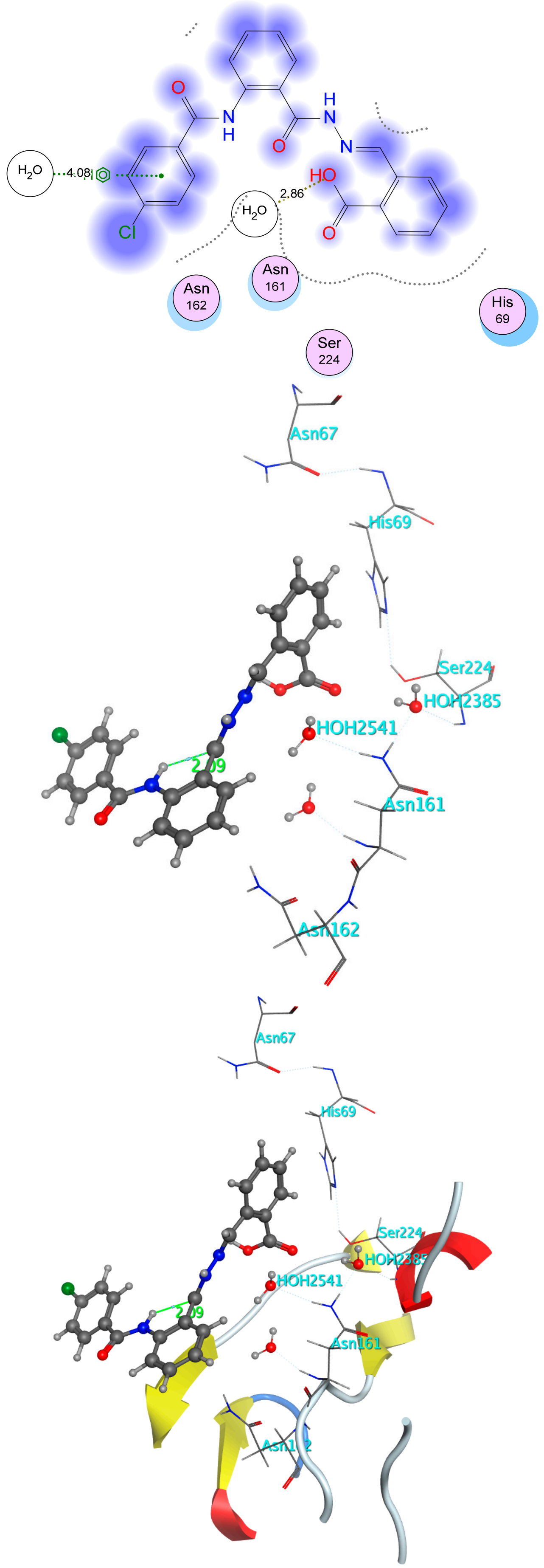

Docking pose for compound **6l** in the active site of Proteinase K

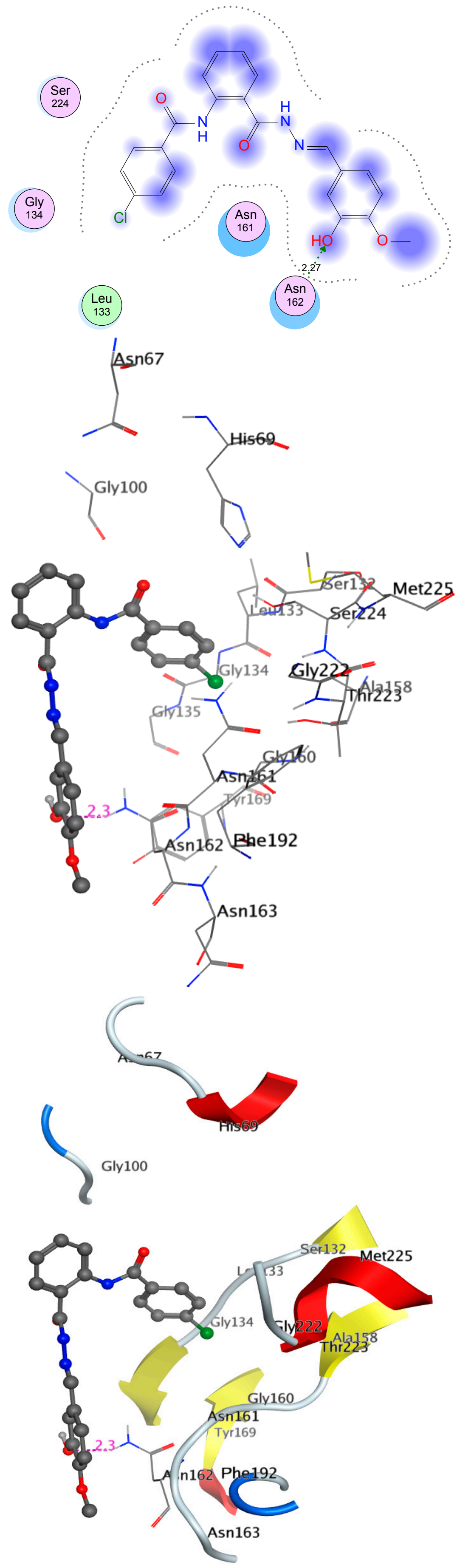

Docking pose for compound **6m** in the active site of Proteinase K

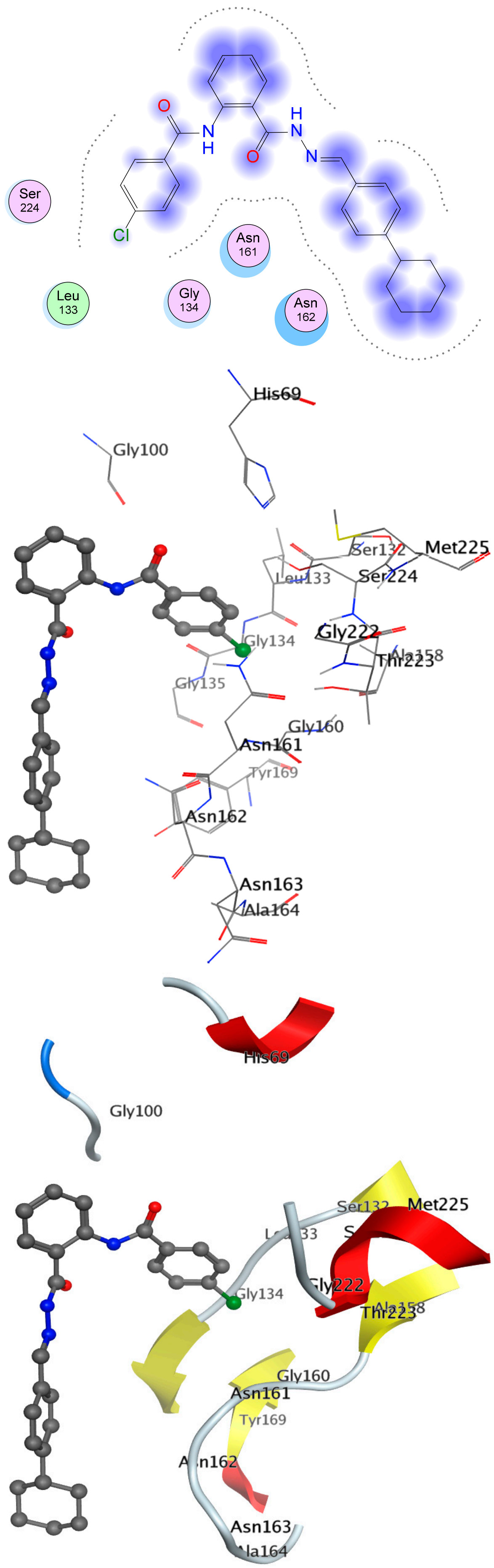

Docking pose for compound **6n** in the active site of Proteinase K

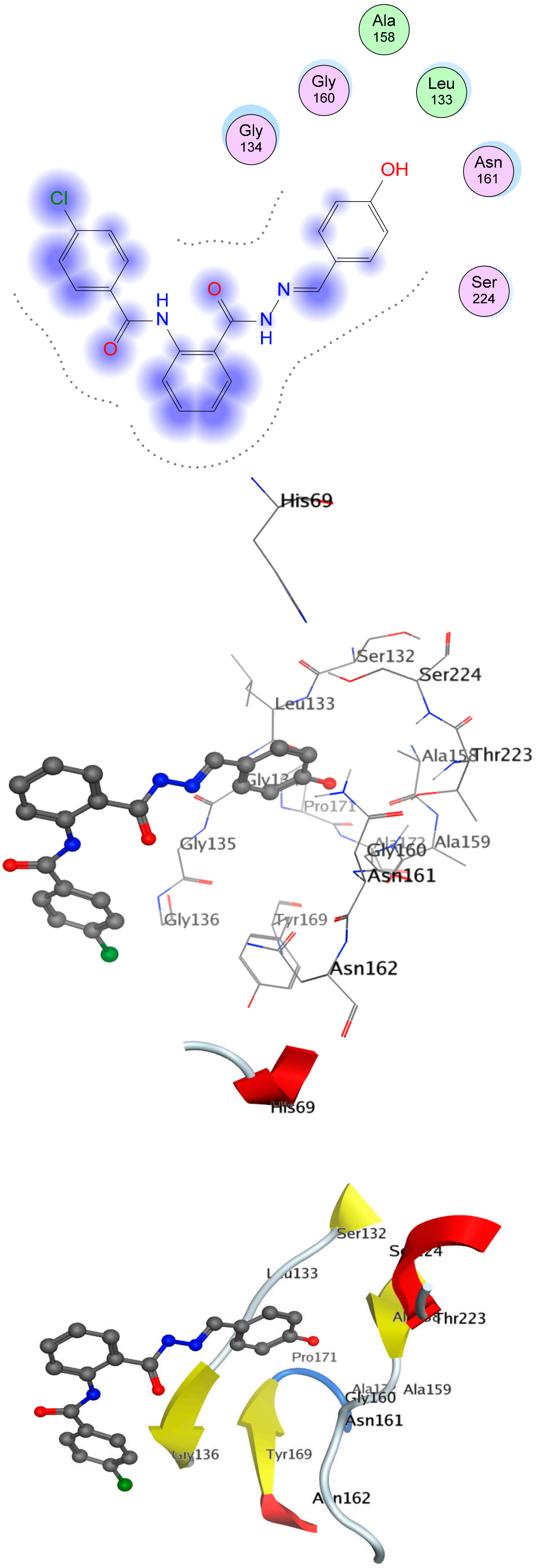

Docking pose for compound **6o** in the active site of Proteinase K

Figures S2. Docking poses of compounds **6a–o** in active site of Proteinase K.

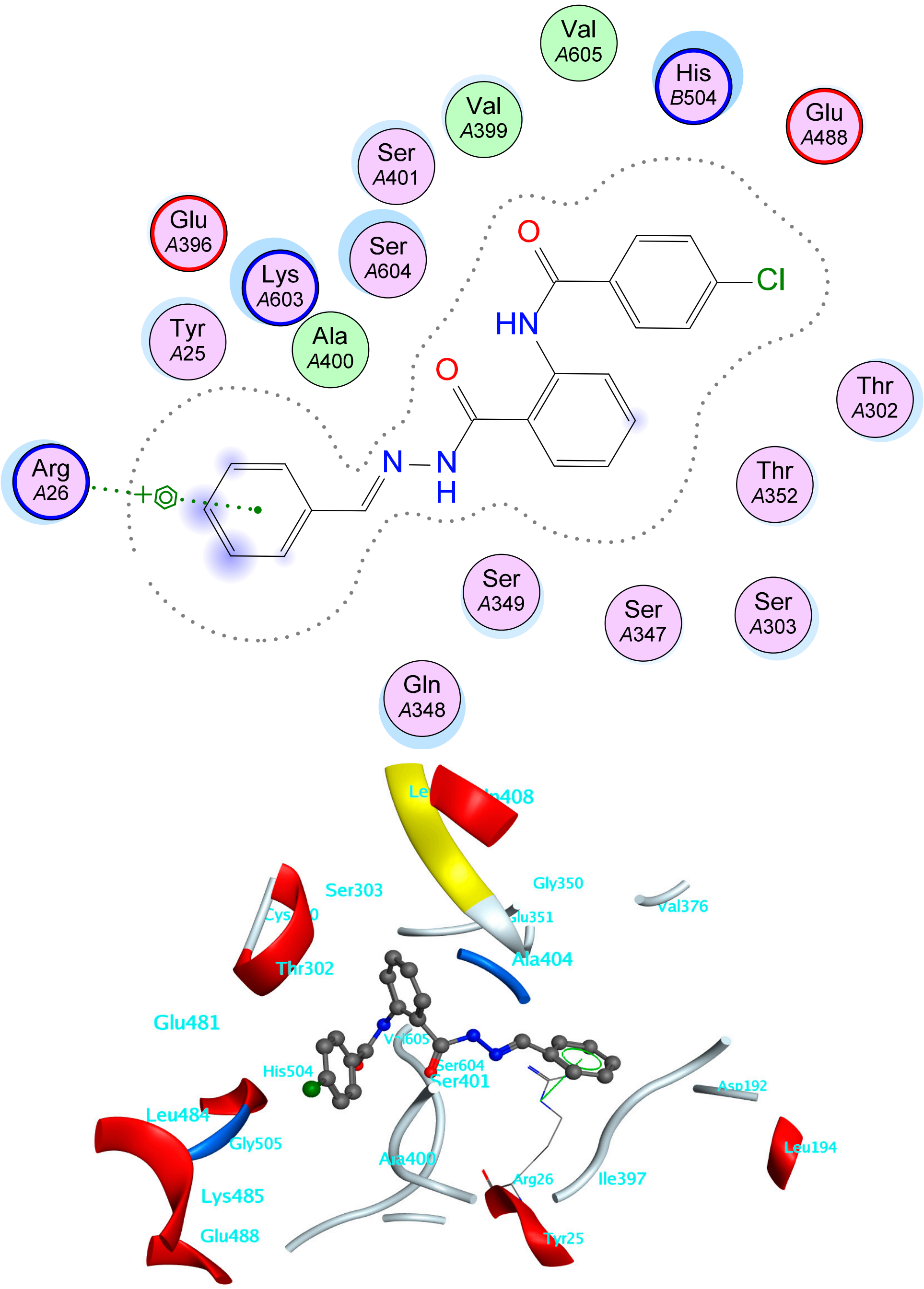

Docking pose in of **6a** in glucosamine-6-phosphate (GlcN6P) synthase

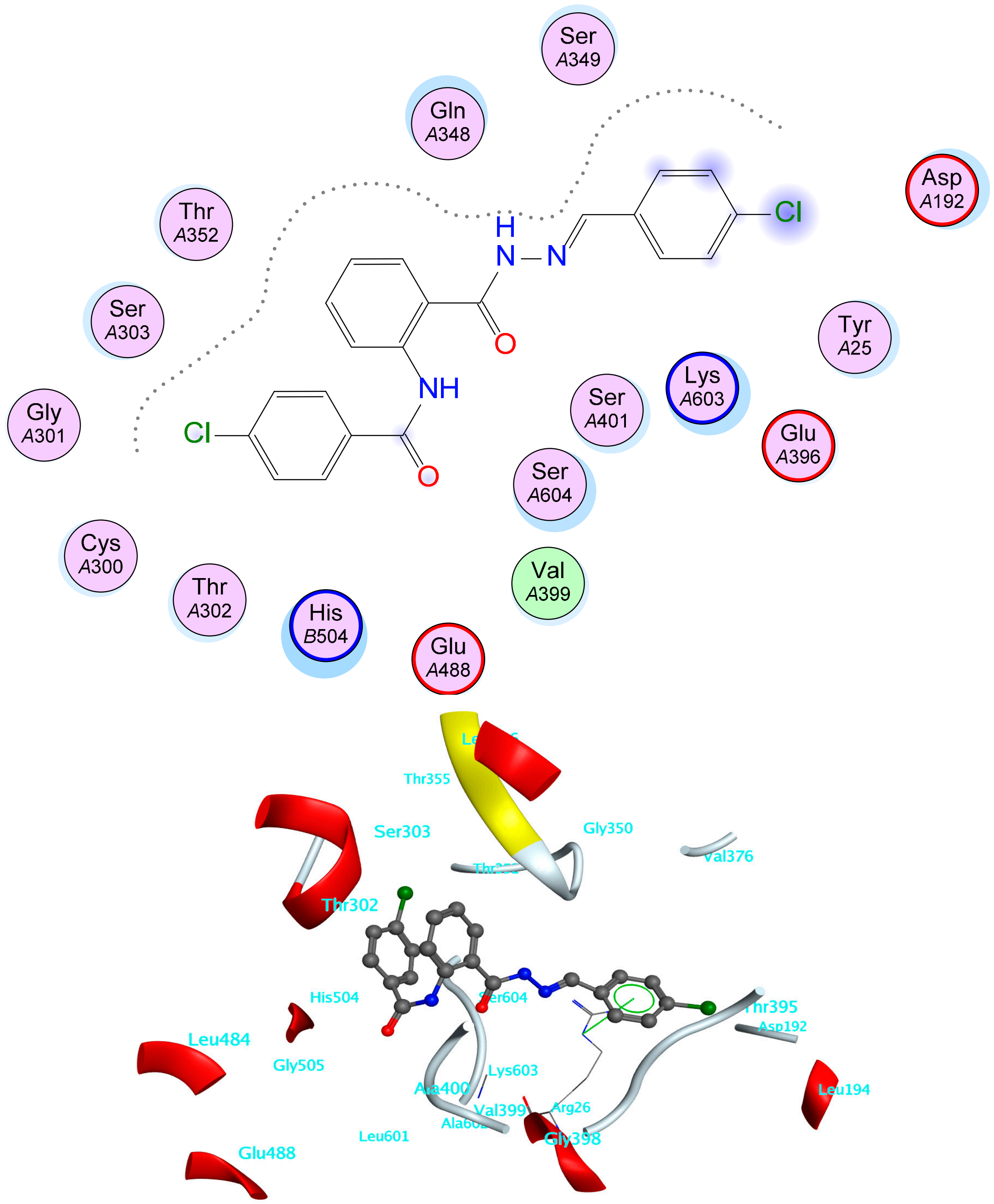

Docking pose in of **6b** in glucosamine-6-phosphate (GlcN6P) synthase

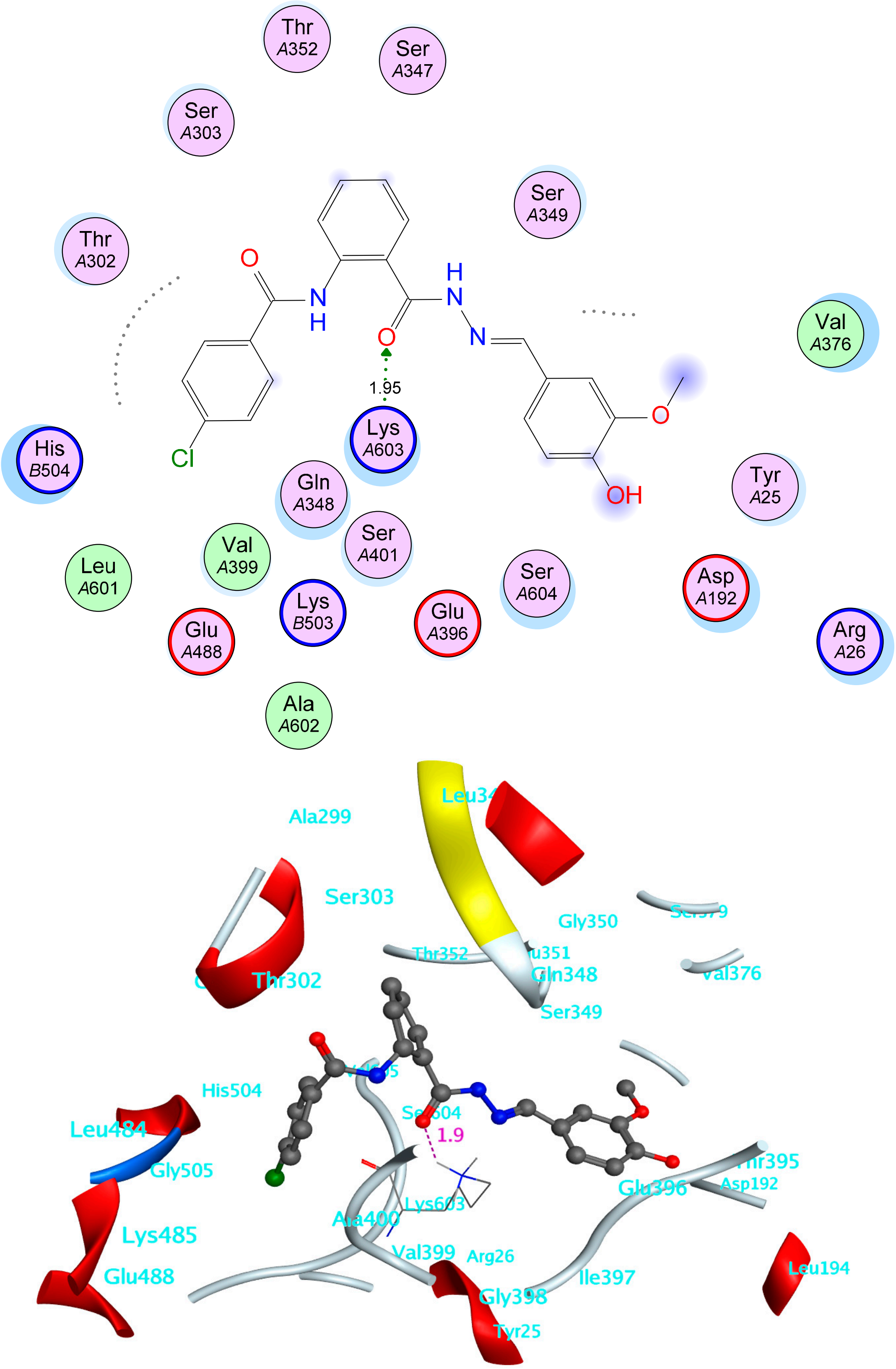

Docking pose in of **6c** in glucosamine-6-phosphate (GlcN6P) synthase

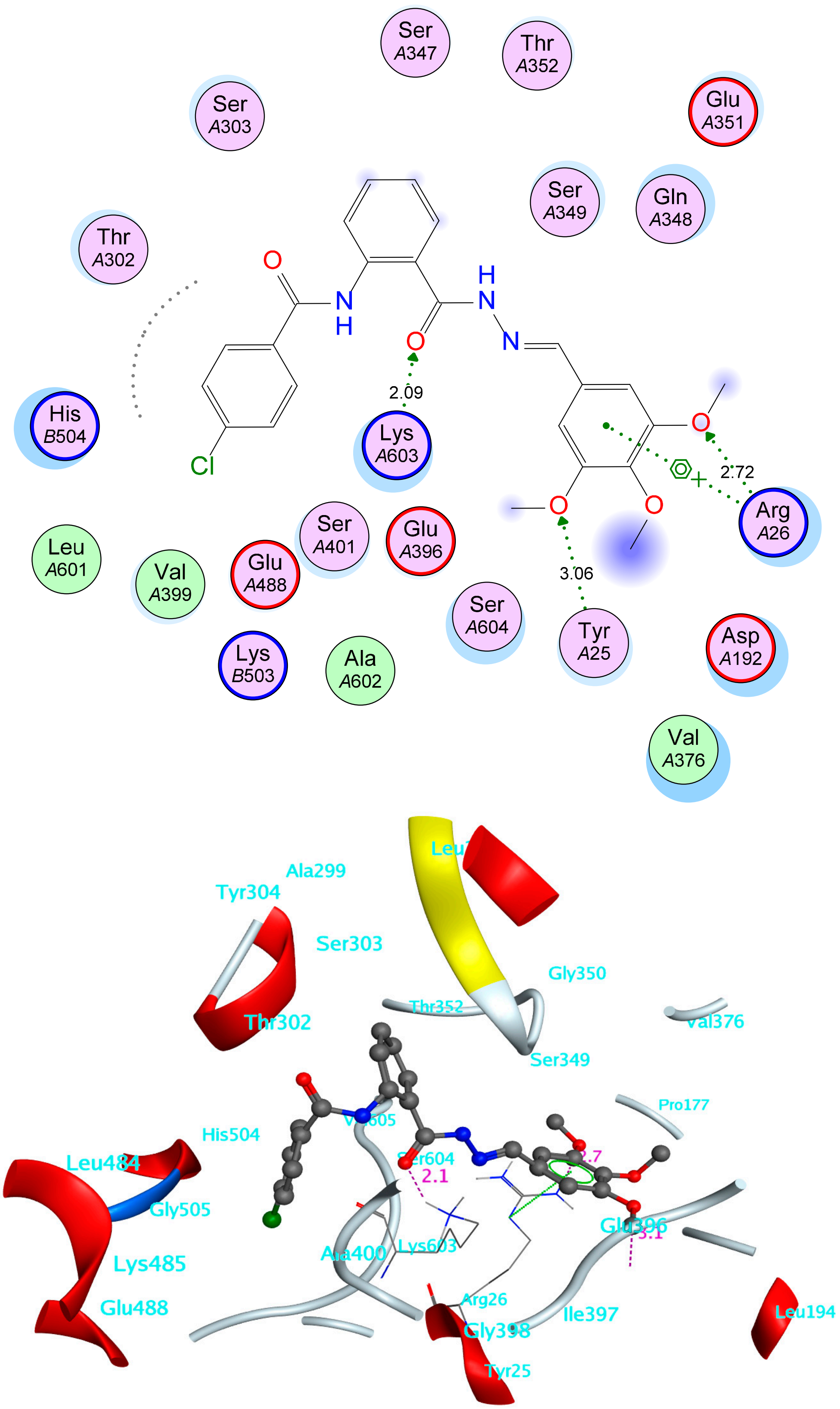

Docking pose in of **6d** in glucosamine-6-phosphate (GlcN6P) synthase

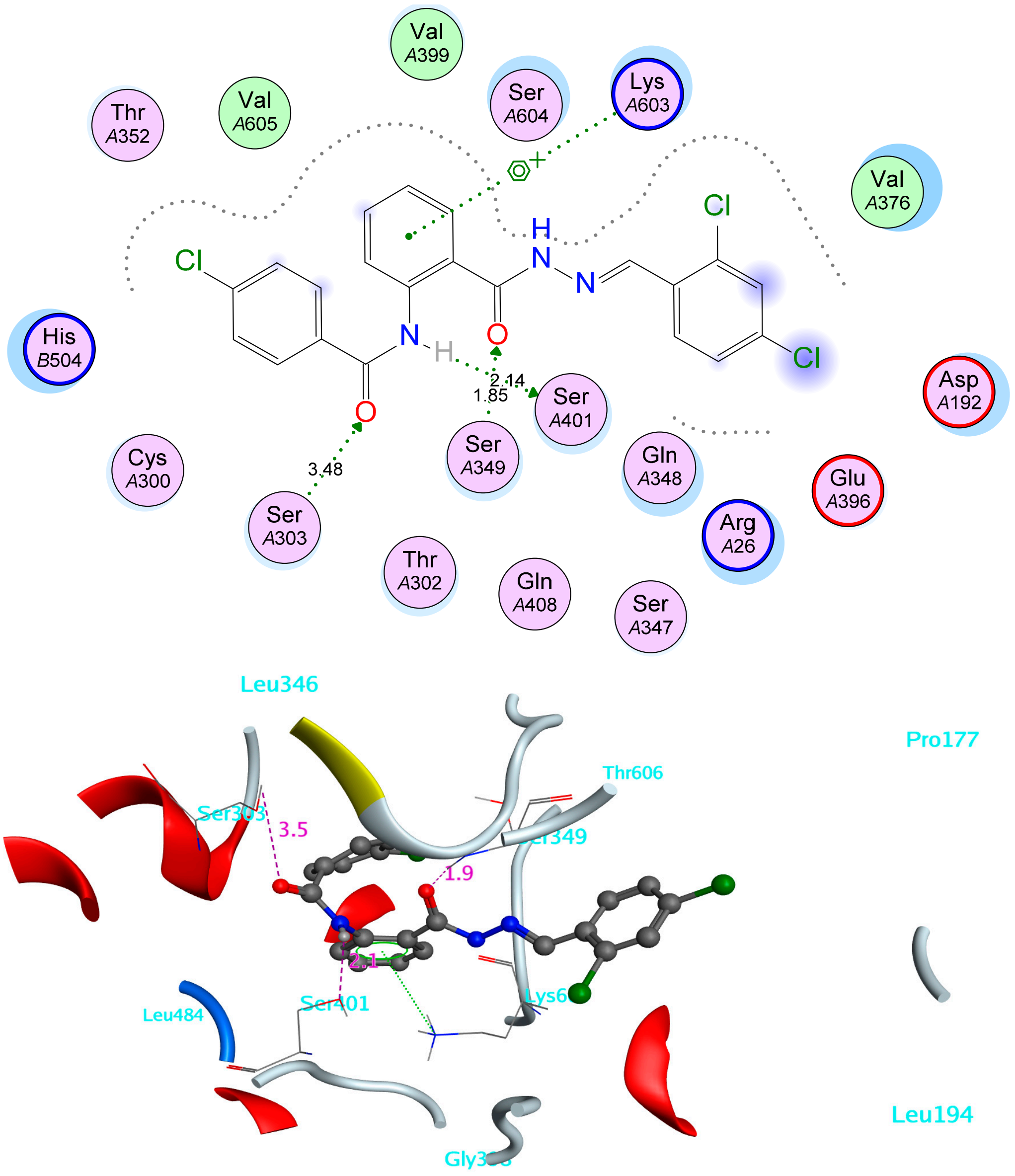

Docking pose in of **6e** in glucosamine-6-phosphate (GlcN6P) synthase

Figures S3. Docking poses of compounds **6a–e** in active site of glucosamine-6-phosphate (GlcN6P) synthase.
